# Transforming NiCo_2_O_4_ nanorods into nanoparticles using citrus lemon juice enhancing electrochemical properties for asymmetric supercapacitor and water oxidation†

**DOI:** 10.1039/d3ra02438e

**Published:** 2023-06-20

**Authors:** Shusheel Kumar, Aneela Tahira, Adeel Liaquat Bhatti, Muhammad Ali Bhatti, Riaz Hussain Mari, Nek Muhammad Shaikh, Muhammad Yameen Solangi, Ayman Nafady, Mélanie Emo, Brigitte Vigolo, Antonia Infantes-Molina, Alberto Vomiero, Zafar Hussain Ibupoto

**Affiliations:** a Institute of Physics, University of Sindh Jamshoro 76080 Sindh Pakistan; b Institute of Chemistry, University of Sindh Jamshoro 76080 Sindh Pakistan zaffar.ibhupoto@usindh.edu.pk; c Institute of Chemistry, Shah Abdul Latif University Khairpur Mirs Sindh Pakistan; d Institute of Environmental Sciences, University of Sindh Jamshoro 76080 Sindh Pakistan; e Mehran University of Engineering and Technology 7680 Jamshoro Sindh Pakistan; f Université de Lorraine, CNRS, IJL F-54000 Nancy France; g Department of Inorganic Chemistry, Crystallography and Mineralogy, Unidad Asociada al ICP-CSIC, Faculty of Sciences, University of Malaga, Campus de Teatinos 29071 Malaga Spain; h Department of Engineering Sciences and Mathematics, Division of Material Science, Luleå University of Technology Luleå Sweden alberto.vomiero@ltu.se; i Department of Molecular Sciences and Nanosystems, Ca' Foscari University of Venice Venezia Mestre Italy; j Chemistry Department, College of Science, King Saud University Riyadh 11451 Saudi Arabia

## Abstract

Recently, the nanostructured nickel–cobalt bimetallic oxide (NiCo_2_O_4_) material with high electrochemical activity has received intensive attention. Beside this, the biomass assisted synthesis of NiCo_2_O_4_ is gaining popularity due to its advantageous features such as being low cost, simplicity, minimal use of toxic chemicals, and environment-friendly and ecofriendly nature. The electrochemical activity of spinel NiCo_2_O_4_ is associated with its mixed metal oxidation states. Therefore, much attention has been paid to the crystal quality, morphology and tunable surface chemistry of NiCo_2_O_4_ nanostructures. In this study, we have used citrus lemon juice consisting of a variety of chemical compounds having the properties of a stabilizing agent, capping agent and chelating agent. Moreover, the presence of several acidic chemical compounds in citrus lemon juice changed the pH of the growth solution and consequently we observed surface modified and structural changes that were found to be very effective for the development of energy conversion and energy storage systems. These naturally occurring compounds in citrus lemon juice played a dynamic role in transforming the nanorod morphology of NiCo_2_O_4_ into small and well-packed nanoparticles. Hence, the prepared NiCo_2_O_4_ nanostructures exhibited a new surface-oriented nanoparticle morphology, high concentration of defects on the surface (especially oxygen vacancies), sufficient ionic diffusion and reaction of electrolytic ions, enhanced electrical conductivity, and favorable reaction kinetics at the interface. The electrocatalytic properties of the NiCo_2_O_4_ nanostructures were studied in oxygen evolution reaction (OER) at a low overpotential of 250 mV for 10 mA cm^−2^, Tafel slope of 98 mV dec^−1^, and durability of 40 h. Moreover, an asymmetric supercapacitor was produced and the obtained results indicated a high specific capacitance of (*C*_s_) of 1519.19 F g^−1^, and energy density of 33.08 W h kg^−1^ at 0.8 A g^−1^. The enhanced electrochemical performance could be attributed to the favorable structural changes, surface modification, and surface crystal facet exposure due to the use of citrus lemon juice. The proposed method of transformation of nanorod to nanoparticles could be used for the design of a new generation of efficient electrocatalyst materials for energy storage and conversion uses.

## Abstract 2

Recently, the nanostructured nickel–cobalt bimetallic oxide (NiCo_2_O_4_) material with high electrochemical activity has received intensive attention.

## Graphical abstract

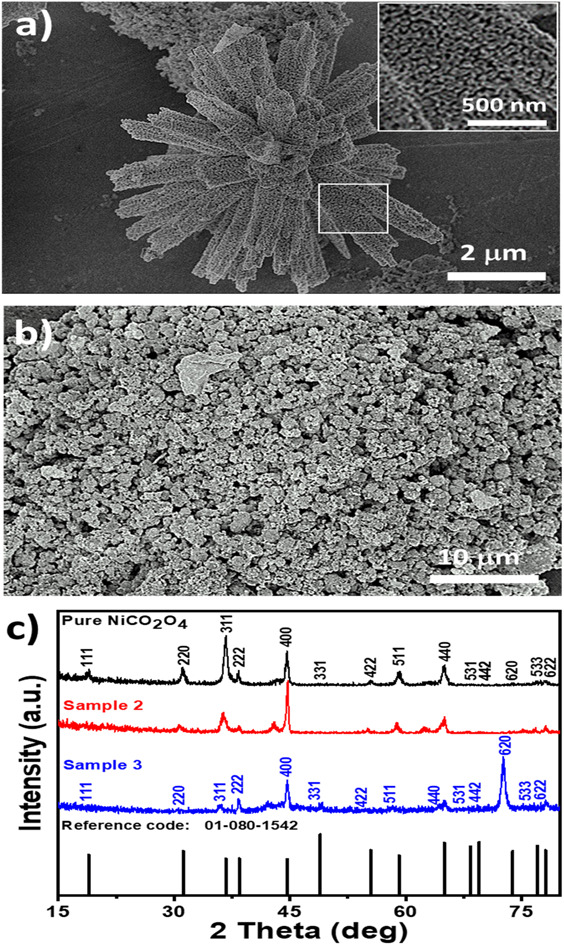

## 1. Introduction

Supercapacitors have been found to be highly effective electrochemical energy storage devices, hence significant advancements have been made in this field.^1–3^ Supercapacitors have advantageous features including a high energy density, long cycling durability, high power density and extended spectrum of operational temperature conditions in comparison to the conventional capacitors and regular electrochemical batteries.^4–6^ Generally, there are two classes of supercapacitors according to their charge–discharge behavior. The first type is the electrochemical double layer capacitors (EDLCs), which store charge on the basis of an electrostatic approach at the active sites of the electrode material *via* reversible adsorption of electrolytic ions. The second type is the pseudo-capacitors or redox supercapacitors, which store the charge using fast and reversible redox processes.^6^ For this reason, the preparation of unique architectures of nanostructured materials is highly desirable for the development of energy storage devices like supercapacitors (SCs). Spinel nickel–cobalt bimetallic oxide (NiCo_2_O_4_) can be seen as a strong candidate for oxygen evolution reaction (OER) and SCs due to its favorable variety of mixed oxidation states, fast charge transfer of electrons and excellent electrical conductivity.^7–9^ It has been shown that the OER and SC activity of NiCo_2_O_4_ is highly dependent on its well oriented shape, for example nanoparticles,^10,11^ microcuboids,^12^ hollow spheres,^13^ octahedrons,^14^ and nanocages.^15^ The combustion of fossil fuels inherently produces a large amount of greenhouse gases causing a global warming effect and ultimately polluting the environment.^16,17^ Hence, the challenges of clean energy and its storage have been taken seriously by developing new functional materials. For the purpose of clean energy, various technologies are used, such as hydropower, solar energy, airstream and biomass based fuel production.^18–20^ Water dissociation into hydrogen gas does not take place without the input of energy due to strong H–O chemical bonding, therefore theoretically a minimum voltage of 1.23 V is needed for the dissociation of water. Such a high energy requirement is difficult to meet using the existing renewable energy sources or fossil fuels; therefore, the synthesis of numerous electrocatalysts has been attempted to lower the energy barrier for water splitting.^13–15^ Typically, the OER and HER reactions take place at the anode and the cathode, respectively.^21^

Among both reactions, OER is very challenging as it involves the transfer of four electrons and is considered as the bottleneck challenge in the exploitation of efficient hydrogen production from water splitting.^22–27^ To this end, the most efficient electrocatalysts are noble metals, like Ru/Ir/Pt/Pd. However, their large scale utilization is limited due to their high scarcity and cost.^21^ To overcome this, low-cost and earth abundant transition metal-based electrocatalysts have been investigated.^28–32^ NiCo_2_O_4_ nanostructures are widely used for OER due to its high catalytic activity and electrical conductivity.^33–35^ However, the performance of NiCo_2_O_4_ is limited by the low density of catalytic sites and sluggish charge transfer rate at the interface of electrode and electrolyte. Furthermore, it has been shown that the electrochemical properties of NiCo_2_O_4_ are highly dependent on its shape, structure and chemical composition.^36,37^ For this reason, an enhanced electrochemical activity of nanoplate-like NiCo_2_O_4_ was observed.^38^ Various factors effect the morphological transformation of nanostructured materials and electrochemical properties, such as pH, temperature, surfactants, and synthetic routes, which have been extensively studied.^39^ Hence, it is a big challenge to prepare different morphologies of NiCo_2_O_4_ for improved electrochemical performances.

The use of new synthetic methodologies has been demonstrated to be effective in increasing the performance of NiCo_2_O_4_ nanostructures.^40,41^ The use of biomass for tuning the catalytic activity of NiCo_2_O_4_ nanostructured materials is considered extensively these days, hence different biomasses have been used during the synthesis.^40,41^ Previous studies have used different biomasses containing phytochemicals and they have modified the surface properties of NiCo_2_O_4_ nanostructures very effectively.^40^ Previously, the utilization of ascorbic acid and citric acid from citrus lemon juice has been explored in the synthesis of a ZnO material and its phase transformation, morphology and optical properties were studied.^42,43^ Citrus lemon juice contains a wide range of unique chemical compounds, as can be seen from ESI Scheme 1,† and their molecular structures were drawn using king draw software. The scientific details of citrus lemons can be listed as Tracheophyta (phylum), Magnoliopsida (class), Sapindales (order), Rutaceae (family), and *Citrus limon*/*limonum* (species). In this study, citrus lemon was employed to alter the pH of the growth solution in addition to acting as a capping, reducing and stabilizing agent during the preparation of a NiCo_2_O_4_ nanostructured material. Moreover, less attention has been paid to the use of citrus lemon juice to create surface oxygen vacancies, achieve high Ni and Co oxidation state ratios, achieve structural transformation, create highly surface-active sites, and lead to high compatibility of the NiCo_2_O_4_ nanostructured material with the electrode, and these aspects of material design have not been studied in the development of energy conversion and storage systems in the existing literature. Importantly, a low cost and simple method for the transformation of nanorods to nanoparticles would provide an advancement in the field of nanoscience for the development of future generations of functional nanostructures for a wide range of applications. Therefore, keeping in mind the natural product chemistry of citrus lemon juice, particularly citric acid, malic acid, and ascorbic acid, we have utilized them together for the first time to enhance the electrochemical performance of NiCo_2_O_4_ nanostructured materials. Furthermore, the creation of terminal oxygenated groups through the use of a variety of citrus lemon juice ingredients opens a new gateway for the synthesis of surface modified nanostructured materials with advanced functional properties. For this purpose, we have studied the effects of these aspects of citrus lemon juice on NiCo_2_O_4_ nanostructured materials towards efficient energy storage and OER applications for the first time.

In this research work, we have used a variety of oxygenated sites of various natural compounds of citrus lemon juice for the hydrothermal preparation of short range and well packed nanoparticles of NiCo_2_O_4_.

## 2. Experimental section

### 2.1. Chemical reagents

Nickel chloride hexahydrate 98% (NiCl_2_·6H_2_O), cobalt chloride hexahydrate 98% (CoCl_2_·6H_2_O), urea 99% (N_2_H_4_CO), potassium hydroxide 90% (KOH), ruthenium oxide 99.9% (RuO_2_), and alumina 5% (Al_2_O_3_) paste (0.3 μM) were of analytical grade and they were purchased from Sigma-Aldrich, Karachi, Sindh, Pakistan. Fresh citrus lemon fruit was purchased from a local market of Jamshoro, Sindh, Pakistan.

### 2.2. Preparation of NiCo_2_O_4_ nanostructured material using citrus lemon juice

Prior to the synthesis of the NiCo_2_O_4_ nanostructured material, the lemon juice was collected from cleaned lemon fruit using a Philips juicer machine. First, a hydrothermal process was carried out to prepare the bimetallic hydroxide phase, then thermal combustion was performed in air to transform the hydroxide phase into the bimetallic oxide. A typical synthesis was initiated as follows: first, the three main precursors of 0.015 M nickel chloride hexahydrate, 0.1 M cobalt chloride hexahydrate, and 0.1 M urea were mixed in 100 mL of deionized water in a beaker, and this was repeated three times. Three beakers containing the main precursors of nickel salt, cobalt salt and urea were then supplemented with 0.5 mL, 1 mL and 2 mL of lemon juice, respectively. They were labeled as sample 1, sample 2 and sample 3, respectively. The fourth beaker containing the growth precursors of nickel salt, cobalt salt and urea was labeled as the pure sample of NiCo_2_O_4_ nanostructured material. The pH of the growth solution without citrus lemon juice was about 6.72 and the pH of the growth solution containing 0.5 mL and 1 mL of lemon juice was about 6.2 and 5.8, respectively. Furthermore, the pH of the growth solution with the addition of 2 mL of lemon juice was found to be about 3.2. The four beakers with the growth solutions were sealed very tightly with aluminum foil and placed in an electric oven for 5 h at 95 °C. After the growth process, the hydroxide product of nickel–cobalt was collected onto filter paper and washed with deionized water. The pH of the post growth solutions of pure NiCo_2_O_4_, sample 1, sample 2, and sample 3 was measured as 6.44, 5.92, 5.28, and 2.97, respectively. Next, the product was dried and subjected to thermal combustion treatment in air for 5 h at 500 °C. Finally, we successfully obtained a black product of NiCo_2_O_4_ nanostructured material named sample 1, sample 2, sample 3 and pure sample.

### 2.3. Structural characterization of the as-prepared NiCo_2_O_4_ nanostructured materials

The characterization of the various NiCo_2_O_4_ nanostructured materials was done with respect to morphology, crystal arrays, chemical composition and surface defects using various analytical techniques. SEM was used to study the morphology of the NiCo_2_O_4_ nanostructures at an applied voltage of 2 kV with a ZEISS Gemini SEM 500 equipped with a field emission gun. Powder X-ray diffraction was employed to identify the phase and purity of the NiCo_2_O_4_ nanostructured material under the experimental conditions of X-rays with a wavelength of *λ*_Kα_ = 1.5406 Å originating from a Cu anode at an applied potential of 45 kV and 45 mA current. Transmission and scanning transmission electron microscopy (TEM and STEM, respectively) observations were made using a JEOL JEM – ARM 200F Cold FEG microscope working at 200 kV and equipped with a probe corrector (Cs). Chemical analyses were done with energy dispersive X-ray (EDX) spectroscopy (SDD, Jeol DRY SD 30 GV). X-ray photoelectron spectroscopy (XPS) with the X-ray source of Al K_α_ at 30 eV was used for the surface chemical composition analysis. The measured binding energies of the as-prepared NiCo_2_O_4_ nanostructured materials were fitted with the reference binding energy of the C 1s peak at 284.6 eV.

### 2.4. Electrochemical characterization of the surface modified NiCo_2_O_4_ nanostructured material

We tested the different NiCo_2_O_4_ samples in supercapacitor and OER half-cell water splitting applications in an aqueous solution of KOH electrolyte. The supercapacitor investigations were performed with a three electrode cell set-up in 3.0 M KOH. The reference electrode was based on the calomel electrode (Hg/HgO), and a graphite rod was used as the counter electrode. The working electrode was the glassy carbon electrode (GCE) with a diameter of 3.0 mm and it was used for the deposition of NiCo_2_O_4_ nanostructured materials using a drop casting approach. Before modification, the GCE was cleaned with 0.3 μM alumina powder slurry and rubbed by silicon paper; consequently it was washed several times with deionized water. The supercapacitor characterization was done with chronopotentiometry (CP) by measuring galvanostatic charge–discharge (GCD) at various current densities for the estimation of specific capacitance (*C*_s_) F g^−1^, cycling stability and energy density. The calculations of the supercapacitor application are shown in S1.†

**Scheme 1:**
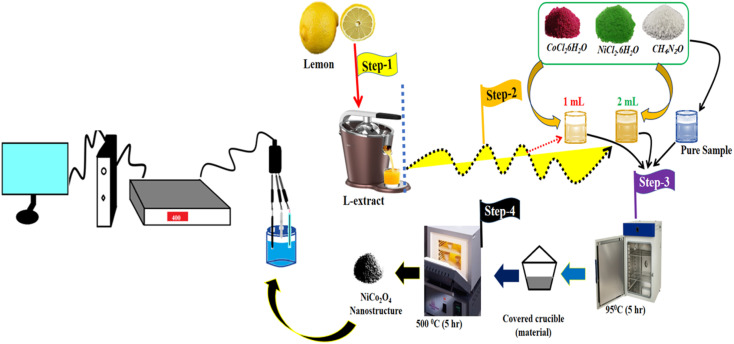
Stepwise preparation of NiCo_2_O_4_ nanostructured material using lemon juice and the electrochemical testing set-up.

Furthermore, OER studies were performed on the different NiCo_2_O_4_ nanostructures in 1 M KOH. The catalyst ink of the NiCo_2_O_4_ nanostructures was prepared by dispersing 5 mg of each sample in 4 mL of deionized water and 100 μL of 5% Nafion. The homogenous dispersion was obtained by using a sonication process for 10 min. Then, 5 μL of each catalyst ink with an approximately mass of 0.2 mg was deposited onto the cleaned GCE using a drop casting method and dried with blown air. The OER half-cell investigations were performed using different electrochemical modes like cyclic voltammetry (CV), linear sweep voltammetry (LSV), chronopotentiometry (CP) and electrochemical impedance spectroscopy (EIS). Prior to LSV measurements, three CV cycles were done at a scan rate of 10 mV s^−1^ for the measurement of stable LSV curves for the OER process at a scan rate of 2 mV s^−1^. EIS was used to measure the kinetics of charge transfer at the interface of modified electrode and electrolyte under the measurement conditions of a sweeping frequency range from 100 kHz to 0.1 Hz using an amplitude of 5 mV and OER onset potential. The CV curves in the non-faradaic region at various scan rates were measured to find the amount of active surface area using electrochemical active surface area (ECSA) calculations. The long-term durability was investigated by using the chronopotentiometry technique for the time period of 40 hours at a constant current density of 20 mA cm^−1^. The measured potential against calomel reference electrodes was converted to reversible hydrogen electrode (RHE) by following the Nernst equation. The Tafel equation was used to find the Tafel slope values from the linear part of the LSV curves for the illustration of reaction kinetics. All the energy storage and conversion measurements were carried out under standard conditions. The stepwise preparation methodology and electrochemical testing are shown in Scheme 1.

## 3. Results and discussion

### 3.1. Growth mechanism of the NiCo_2_O_4_ nanostructured material in the presence of citrus lemon juice

**Scheme 2:**
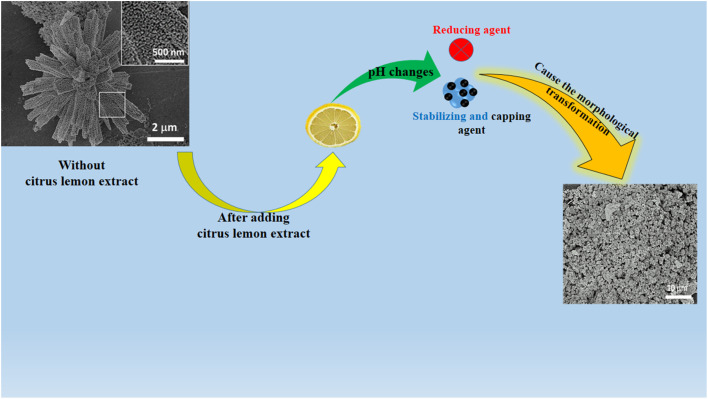
Growth mechanism for the transformation of NiCo_2_O_4_ nanorods to nanoparticles.

Generally in the growth process of NiCo_2_O_4_, the primary use of urea is to add hydroxyl ions into the growth solution to favor the formation of the hydroxide phase of nickel–cobalt. Moreover, urea provides the hydroxyl ions through the release of ammonia, which further reacts with the water and gives out hydroxyl ions for binding with metallic ions coming from their respective salt precursors. This led to the formation of a nanorod-like morphology, as shown in Scheme 2. Whereas, the purpose of adding citrus lemon is to change the pH of the growth solution due to the presence of the several relatively acidic compounds like ascorbic acid, malic acid and citric acid. At the same time, citrus lemon provided capping, reducing and stabilizing agents during the preparation of the NiCo_2_O_4_ nanostructured material. The significant variation in the pH of the growth solutions with 0.5 mL, 1 mL and 2 mL of lemon juice could play a dynamic role in transforming the nanorods into nanoparticles, as shown in Scheme 2.

### 3.2. Physical characterization of as-prepared different NiCo_2_O_4_ nanostructures

**Fig. 1:**
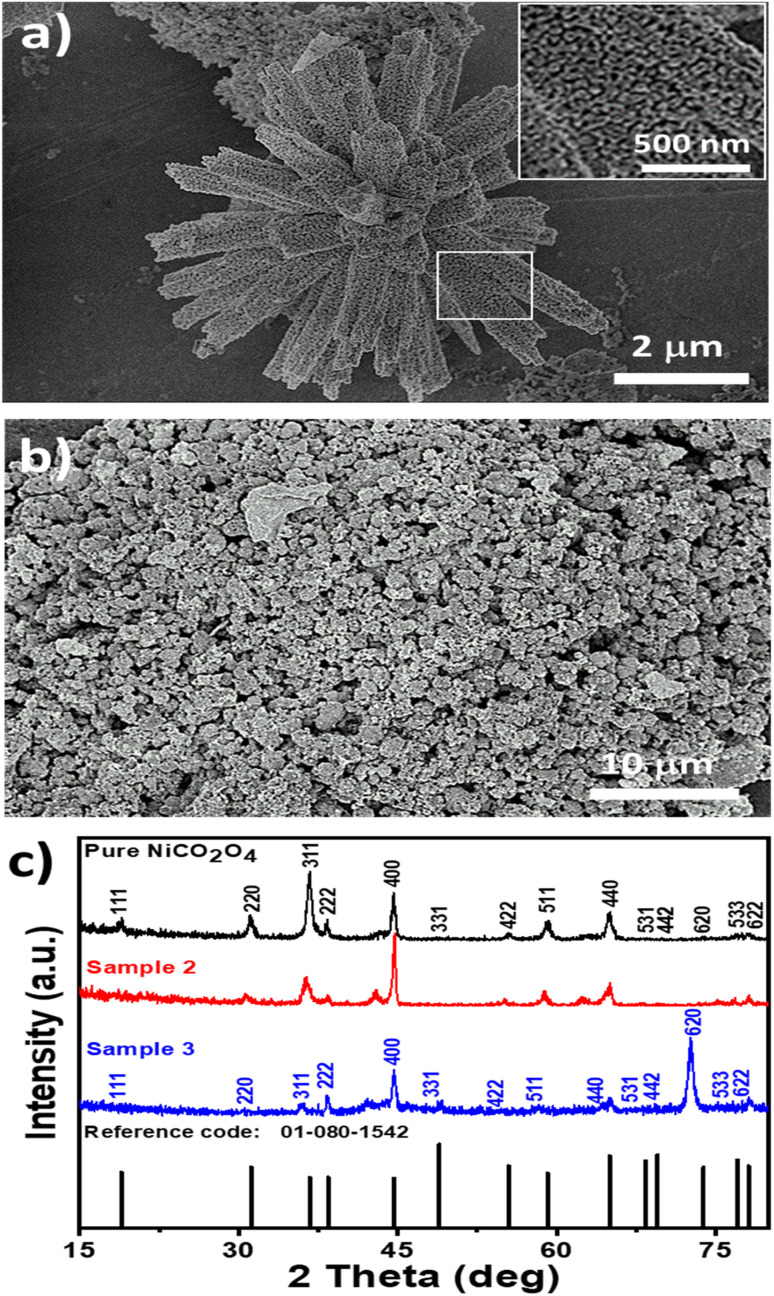
Distinctive SEM images at different magnifications of (a) pristine NiCo_2_O_4_ nanostructures and (b) NiCo_2_O_4_ nanostructures prepared with 1 mL of lemon juice (sample 2) and (c) XRD patterns of pristine NiCo_2_O_4_ nanostructures and (b and c) NiCo_2_O_4_ nanostructures prepared with 1 mL (sample 2) and 2 mL of lemon juice (sample 3).

The morphology and structure of the synthesized NiCo_2_O_4_ based nanocatalysts were studied using SEM and XRD, respectively (Fig. 1). Fig. 1a shows the typical shape orientation of the pristine NiCo_2_O_4_ nanostructures prepared by a hydrothermal process, which is characterized by a nanorod-like morphology with a length of several microns and average diameter in the range of 300–500 nm. The nanorod is assembled by nanoparticles with a dimension of below 100 nm, as shown in Fig. 1a, inset. Furthermore, the morphology of the citrus lemon juice assisted NiCo_2_O_4_ nanostructures with a volume of 1 mL (sample 2, Fig. 1b) was found to be different from the pure sample of NiCo_2_O_4_ nanostructured material. The nanorod morphology was transformed into well packed small size nanoparticles. The size of the small nanoparticles was below 100 nm, which is a typical nanostructured phase of as-prepared NiCo_2_O_4_ nanostructures. The growth process in the presence of citrus lemon juice can be proposed in terms of the oxygenated terminal of the various natural compounds present in the citrus lemon juice. These natural product molecules can act as a stabilizing agent, capping agent, and chelating agent, which played an important role in the transformation of nanorod morphology into nanoparticles. Moreover, the pH of the growth solution was changed due to the presence of ascorbic acid, citric acid, and malic acid and also their stabilizing, capping and chelating properties resulted in the formation of nanoparticles, as can be seen in Fig. 1b. Thus, the compact nature of the nanoparticles with uniform electron transfer during the electrochemical reaction can contribute to the durability of the material for long term applications. Citrus lemon juice is naturally rich with acidic compounds with a high density of terminated oxygen groups in each molecule for transforming nanorods to nanoparticles. The significant variation in the pH of growth solutions with 0.5 mL, 1 mL and 2 mL of lemon juice could play a dynamic role in altering the structure and surface properties of the nanostructured material. Due to the acidic nature of ascorbic acid, malic acid and citric acid, they had an influence on the pH of the growth solution, and consequently a structural transformation and surface modifications were significantly observed. However, the primary use of citrus lemon juice was to alter the pH of the growth solution and to induce a significant effect on the structure and surface properties of the material. We also noticed that the relative amount of nanostructured material formation was low when 2 mL of citrus lemon juice was used because of the decreased amount of hydroxyl ions that slowed down the growth kinetics. From SEM observations, it is clear that the presence of lemon juice significantly alters the morphology (Fig. 1a and b, with and without lemon juice, respectively). The rod-like shape is completely lost when lemon juice is used in the catalyst preparation and the obtained catalysts have a powdered disordered structure. Tiny particles are still detectable, as confirmed by XRD analysis of crystallite size.

The crystalline phase was studied using the powder XRD technique and the recorded diffraction patterns of samples 2 and 3 are shown in Fig. 1c. The diffraction pattern of sample 1 prepared with 0.5 mL of citrus lemon juice is shown ESI Fig. S1.† The diffraction patterns of the samples of NiCo_2_O_4_ nanostructures showed crystal planes of 111, 220, 311, 222, 400, 422, 511, 531, 442, 533, 620 and 622 and they matched well with the cubic phase of NiCo_2_O_4_. The measured diffraction patterns of the NiCo_2_O_4_ nanostructures were in good agreement with the standard JCPDS card (01-080-1542). Interestingly, the increasing volume of citrus lemon juice reduced the intensity of many of the diffraction peaks due to the variation in the pH of the growth solution, suggesting the dynamic role of citrus lemon juice in the alteration of shape of the NiCo_2_O_4_ nanostructured material. The pattern of sample 2 suggests preferential growth along the 400 direction due its strong reflection intensity and this offered a favorable crystal orientation for high exposure of catalytic sites and swift charge transfer in electrochemical applications. This is the point of difference between sample 2 and the pure NiCo_2_O_4_ nanostructures and sample 3. The XRD analysis confirmed the presence of only the spinel structure with a cubic phase of NiCo_2_O_4_ nanostructures and the preparation of a high quality nanostructured material. Moreover, an enhanced peak for the 620 crystal plane of the NiCo_2_O_4_ nanostructures in the case of sample 3 indicated preferential growth along this direction and this might change the crystal geometry of the NiCo_2_O_4_ nanostructures that may not favor the electrocatalytic activity. The average crystallite size was calculated by using the Scherrer equation and the calculated values are given in Table S1.† It is obvious that 1 mL (sample 2) and 2 mL (sample 3) of lemon juice did not have any substantial influence on the average crystallite size of the NiCo_2_O_4_ nanostructures in comparison to the pure sample. A slight increase in average crystallite size was recorded for sample 3.

**Fig. 2:**
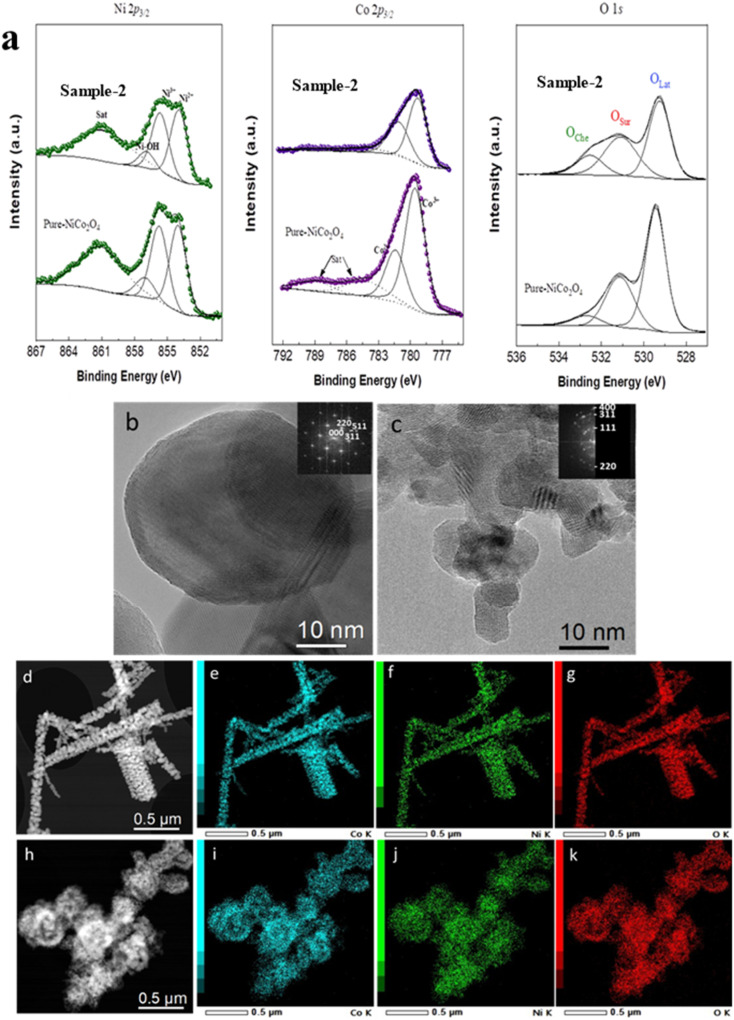
Ni 2p_3/2_, Co 2p_3/2_ and O 1s spectra of pure NiCo_2_O_4_ and NiCo_2_O_4_ nanostructures grown with 1 mL of citrus lemon juice (sample 2) (a). HRTEM micrographs with corresponding Fast Fourier Transform (FFT) (inset) of pure NiCo_2_O_4_ (b) and citrus lemon juice assisted grown NiCo_2_O_4_ nanostructures (c). STEM High Angle Annular Dark Field (HAADF) images of pure NiCo_2_O_4_ (d) and citrus lemon juice assisted grown NiCo_2_O_4_ nanostructures (h) and corresponding X-maps of cobalt (e and i), nickel (f and j) and oxygen (g and k).

To gain deeper insight on the chemical states of metallic ions and surface vacancies of the pure NiCo_2_O_4_ and sample 2, XPS analysis was performed, as shown in Fig. 2a. The photoelectron contributions for the Co 2p_3/2_ signal of pure NiCo_2_O_4_ were observed at 779.5 (Co^3+^), 781.4 (Co^2+^), 784.2 (satellite) and 788.9 (satellite) eV, which is in good agreement with the reported works.^44–46^ Similarly, the Ni 2p_3/2_ contributions were located at different binding energies of 854.0 (Ni^2+^), 855.7 (Ni^3+^), 857.0 (Ni–OH) and 861.1 (satellite) eV.^47^ The relative ratio of Ni^2+^/Ni^3+^ ions and Co^2+^/Co^3+^ ions in pure NiCo_2_O_4_ was found to be 1.13 and 0.44, respectively. For the citrus lemon juice assisted NiCo_2_O_4_ nanostructures (sample 2), the highly resolved spectrum of Co 2p_3/2_ was also fitted, and the corresponding peaks of Co^3+^ and Co ^2+^ were found at binding energies of 779.3 and 781.1 eV, respectively, as shown in Fig. 2.^48^ For the Ni 2p_3/2_ signal, the fitting showed contributions at various binding energies of 854.0 (Ni^2+^), 855.7 (Ni^3+^), 856.1 (Ni–OH) and 860.8 (satellite). The relative ratio of Ni^2+^/Ni^3+^ ions and Co^2+^/Co^3+^ ions for sample 2 was approximately 1.18 and 0.66, respectively. This indicated a greater proportion of reduced species on the surface of sample 2 compared to pure NiCo_2_O_4_. Moreover, the Ni/Co ratio of pure NiCo_2_O_4_ of 1.02 changed to 1.37 in the case of sample 2. In this regard, the XPS analysis revealed that pure NiCo_2_O_4_ and sample 2 feature coexisting Ni^2+^/Ni^3+^ ions and Co^2+^/Co^3+^ ions on their surface and the presented results are strongly supported by a reported work.^49^ Finally, the O 1s spectra for the pure NiCo_2_O_4_ and sample 2 show three essential contributions at *ca.* 529.5 eV, 531.15 eV, and 532.6 eV due to O_Lat_, O_Sur_, and O_Che_, respectively. O_Lat_ represents lattice oxygen in a metal–oxygen structure, O_Sur_ is usually assigned to surface defects and oxygen vacancies, and O_Che_ is assigned to chemisorbed oxygen, as revealed by previous studies.^44^ By quantifying the different oxygen species in both samples, the O_Sur_/O_Lat_ ratio changes from 0.52 to 0.76 from pure NiCo_2_O_4_ to sample 2, indicating that there is a greater proportion of surface species in the latter sample that could play an important role in the OER reaction. The detailed XPS peak attributions for pure NiCo_2_O_4_ and sample 2 are given in Table S2.†

The morphological, crystalline and chemical composition investigations of pristine and citrus lemon juice assisted NiCo_2_O_4_ samples were performed using scanning transmission electron microscopy (STEM) coupled with energy dispersive X-ray spectroscopy (EDXS), high resolution transmission electron microscopy (HRTEM) and selective area electron diffraction (SAED). The pristine NiCo_2_O_4_ sample is mainly composed of particles of diameter between 20 and 80 nm assembled into nanorod-like structures (Fig. 2b and d), while citrus lemon juice assisted NiCo_2_O_4_ is composed of aggregates of nanoparticles of less than 20 nm (Fig. 2c and h). Similar morphological features were observed during SEM analysis. The aggregation of nanoparticles (sample 2) could be ascribed to the collective attraction between nanoparticles through chemical interactions or van der Waals forces. EDXS experiments confirmed the presence of Ni, Co, and O for both samples, without any impurities (Fig. 2d–k). The SAED patterns of pure NiCo_2_O_4_ and citrus lemon juice assisted NiCo_2_O_4_ (Fig. S2†) present rings at 4.7, 2.9, 2.4 and 2.0 Å, corresponding to the *Fd*3̄*m* cubic structure of NiCo_2_O_4_ (*a* = 8.1 Å), as found in XRD experiments. The overall observations on both samples suggest that citrus lemon juice has a major role in transforming the nanorods of pure NiCo_2_O_4_ into nanoparticles.

For a better view of the structural information, various TEM images are shown in Fig. S3,† suggesting the effect of citrus lemon juice on the morphology compared to the material prepared without the citrus lemon juice. The TEM analysis confirms the results from SEM: addition of citrus lemon juice inhibits the formation of a rod-shaped structure of self-assembled nanoparticles, and leads to a homogeneous distribution of tiny nanoparticles, roughly hierarchically clustered in sub-micrometer sized spheres. Also, the *d*-spacing was calculated by using HRTEM micrographs of citrus lemon juice assisted juice NiCo_2_O_4_ and pure NiCo_2_O_4_, as shown in Fig. S4.†

### 3.3. Electrochemical performance evaluation of NiCo_2_O_4_ nanostructures for energy storage applications

**Fig. 3:**
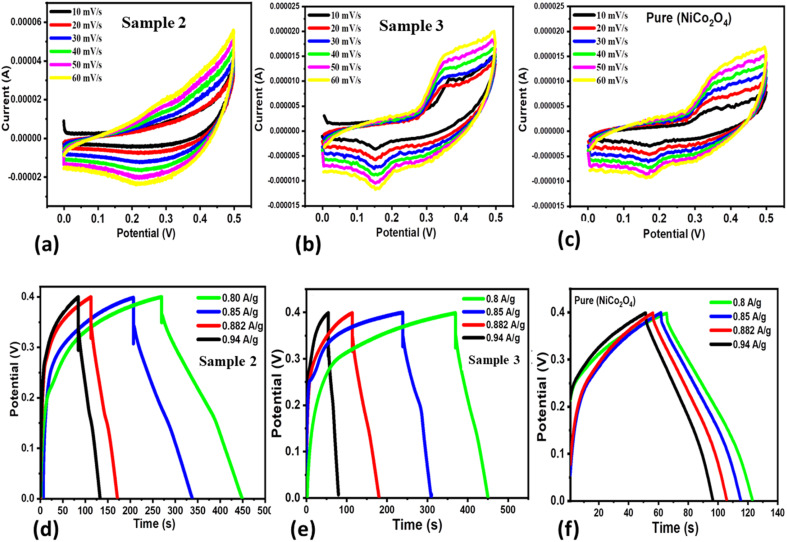
(a–c) Various CV curves at different scan rates for NiCo_2_O_4_ (sample 2), NiCo_2_O_4_ (sample 3), and the pure NiCo_2_O_4_ nanostructures in 3.0 M KOH aqueous electrolytic solution. (d–f) Corresponding GCD curves at various current densities of 0.8, 0.85, 0.88, and 0.94 A g^−1^ recorded in 3.0 M KOH solution.

**Fig. 4:**
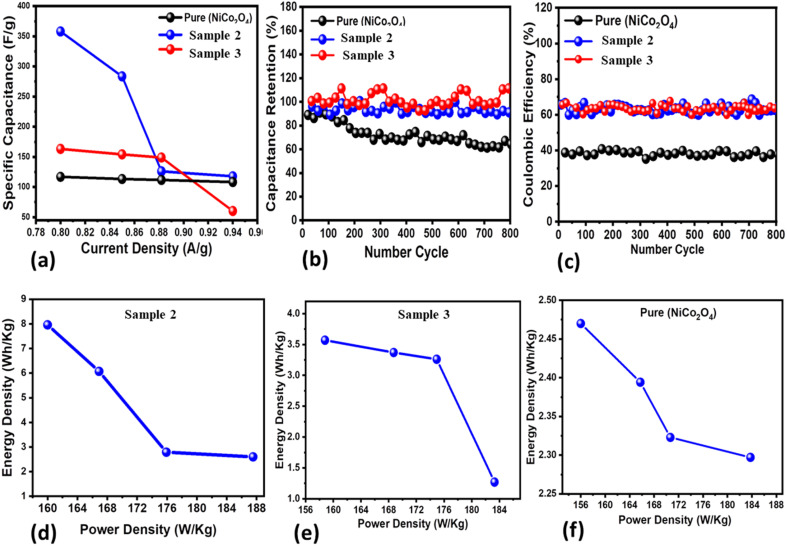
(a) Specific capacitance (*C*_s_) of the pure NiCo_2_O_4_ nanostructures, sample 2 and sample 3 from the GCD curves, (b) percentage specific capacitance retention of the pure NiCo_2_O_4_ nanostructures, sample 2, and sample 3 from the cycling stability of GCD curves, (c) coulombic efficiency of the pure NiCo_2_O_4_ nanostructures, sample 2 and sample 3 from the cycling stability of GCD curves, and (d–f) corresponding energy density of the pure NiCo_2_O_4_ nanostructures, sample 2 and sample 3.

Three new samples of NiCo_2_O_4_ nanostructures (sample 1, sample 2, sample 3) and the pure NiCo_2_O_4_ nanostructures as a reference were prepared to understand the role of citrus lemon juice in tuning the electrocatalytic behavior of the materials. The electrochemical performance was evaluated towards the development of SCs. The prepared nanostructured NiCo_2_O_4_ materials were characterized by using cyclic voltammetry (CV) and galvanostatic charge–discharge (GCD), as shown in Fig. 3. Both the CV and GCD curves were measured with a three electrode cell set-up in an electrolytic environment of 3.0 M KOH aqueous solution, as shown in Fig. 3a–f. The CV characterization uncovered the pronounced redox behavior of the three materials at various scan rates ranging from 10 to 60 mV s^−1^, as shown in Fig. 3a–c. The area of each curve was enhanced with increasing scan rate. Moreover, the shape of the CV curves did not alter with increasing scan rate, suggesting that the prepared materials could deliver superior electrochemical performance. The capacitance from CV was observed to be higher for sample 2 compared to sample 3 and the pure NiCo_2_O_4_ nanostructures at a low scan rate. Beside this, the GCD curves of these materials were measured at different current densities ranging from 0.8 to 0.94 A g^−1^ and their corresponding electrochemical behavior is shown in Fig. 3d–f. It is obvious that sample 2 exhibited the longest discharge time in comparison to sample 3 and the pure NiCo_2_O_4_ nanostructures, as shown in Fig. 3e. Furthermore, the specific capacitance (*C*_s_) values were calculated at 0.8 A g^−1^ for sample 2, sample 3 and the pure NiCo_2_O_4_ nanostructures as 358 F g^−1^, 163 F g^−1^, and 117 F g^−1^, respectively, as shown in Fig. 4a. The cyclic stability was also measured at 0.8 A g^−1^ for the three samples and the corresponding retention efficiency of *C*_s_ was found to be 100–94%, 108–83%, and 90–61%, for sample 2, sample 3 and the pure sample, respectively, as shown in Fig. 4b. After 900 cycles, the retention capacitance was found to be highest for sample 1. It was verified that sample 2 exhibited enhanced cyclic stability with the highest retention; hence, it could be considered as an optimized electrode material for the development of real energy storage devices. The coulombic efficiency of sample 2, sample 3 and the pure sample was observed to be 70%, 64%, and 43%, respectively, as shown in Fig. 4c. The energy density was also estimated at 0.8 A g^−1^ for sample 2, sample 3 and the pure sample of NiCo_2_O_4_ nanostructures as 7.96 W h kg^−1^, 3.57 W h kg^−1^, and 2.47 W h kg^−1^ respectively. The power densities were also calculated for sample 2, sample 3 and the pure NiCo_2_O_4_ nanostructures with corresponding values of 160.00 kW kg^−1^, 158.80 kW kg^−1^, and 156.00 kW kg^−1^, respectively, as shown in Fig. 4d–f. The evaluated electrochemical activity of the as-prepared NiCo_2_O_4_ nanostructures (sample 2) and pure NiCo_2_O_4_ nanostructures for SCs is shown in Table S3.†

For three electrode supercapacitor application, the cyclic stability of sample 1 is either equal or higher to many of the recently reported electrode materials, as given in Table S4.† Many of the materials reported in Table S4† are complex in nature and involve hybrid systems that apparently have excellent performance. However, our presented material synthesis approach is facile, low cost, ecofriendly and environment friendly and our material has comparable specific capacitance to the previous studies, hence offering an alternative with a promising green synthetic strategy for the scale up synthesis of nanostructured materials. The citrus lemon juice changed the pH of the growth solution and this influenced the structural transformation, surface modification and the exposure of favorable crystal facets for enhanced electrochemical performance. The performance of our material can be enhanced compared to the existing literature on supercapacitors by high loading of the proposed material onto a large surface area electrode. Despite the use of a low current density of 0.8 A g^−1^ and narrow potential window, we obtained a considerable high energy density and specific capacitance using the newly prepared NiCo_2_O_4_ nanostructures (sample 2).

Before the development of a two electrode asymmetric supercapacitor device, we evaluated the supercapacitor properties of activated carbon using a three electrode cell configuration and the measured performance is provided in ESI Fig. S5.† First, GCD curves were obtained at various current densities of 0.8, 0.85, 0.88 and 0.94 A g^−1^, then a specific capacitance of 247 F g^−1^ and energy density of 5.47 W h kg^−1^ were calculated, as shown in ESI S5.† The analyzed results of the GCD curves indicated the poor performance of activated carbon (AC).

The practical potential of the NiCo_2_O_4_ nanostructures (sample 2) was also ascertained by developing an asymmetric supercapacitor (ASC) using an anode of the NiCo_2_O_4_ nanostructures (sample 2) and activated carbon as the cathode electrode in aqueous 3.0 M KOH solution. The ASC was represented by (NiCo_2_O_4_ sample 2//AC ASC).

The CV curves of sample 2 and AC in the potential range of −0.2 to 1.5 V are shown in ESI Fig. S5.† It was assumed that the operating potential window of (NiCo_2_O_4_ sample 2//AC ASC) could be enlarged to 1.45 V and this potential window was selected according to published work.^50–54^ Further increasing the potential range could cause the H_2_/O_2_ evolution reaction as previously reported.^50,51^ Hence, the optimum operational potential window was kept approximately to 1.45 V to examine the electrochemical activity of the asymmetric device (NiCo_2_O_4_ sample 2//AC ASC) and the corresponding CV curves are provided in ESI Fig. S6.† The CV results revealed that the overall phenomenon of capacitance of (NiCo_2_O_4_ sample 2//AC ASC) arose from double-layer capacitance and the battery like aspects of the newly prepared spinel bimetallic oxide material. From the CV curves, it is clear that there is no change of shape of CV, indicating the swift charge–discharge features of the (NiCo_2_O_4_ sample 2//AC ASC) device. The corresponding GCD curves of (NiCo_2_O_4_ sample 2//AC ASC) were also measured, as shown in ESI Fig. S6,† for the current density range of 0.8 to 0.94 A g^−1^ and the derived different calculation values of ASC characterization are provided in ESI Fig. S6.† The capacitance retention for 900 cycles was found to be around 64–52%, which is still far better than many of the reported works on bimetallic oxides in the literature, as shown in ESI Fig. S6.† The specific capacitance (*C*_s_) at 0.8 A g^−1^ was calculated as 1519 F g^−1^ and even at 0.94 A g^−1^, specific capacitance was found to be as high as 1459 F g^−1^, as shown in ESI Fig. S6.† The estimated energy density of 33.08 W h kg^−1^ is relatively higher than the reported works with a power density of 633 W kg^−1^, confirming a significant advancement in the field of ASC devices, as shown in ESI Fig. S6.† The coulombic efficiency of 100–84% for the presented ASC system is also excellent, as shown in ESI Fig. S6.† A power density of 633 W kg^−1^ reflects that (NiCo_2_O_4_ sample 2//AC ASC) can maintain an energy density of 33.1 W h kg^−1^, suggesting that these values are still impressive compared to the already existing NiCo_2_O_4_ based ASC systems such as the composite system of NiCo_2_O_4_@MnO_2_ hybrid//AC (an energy density of 37.8 W h kg^−1^ at a power density of 187.5 W kg^−1^),^52^ N-doped carbon-coated NiCo_2_O_4_//AC (29.4 W h kg^−1^ at the power density of 349 W kg^−1^),^55^ HDC@NiCo_2_O_4_@PPy//HDC@NiCo_2_O_4_@PPy (17.5 W h kg^−1^ at the power density of 500 W kg^−1^),^50^ NiCo_2_O_4_ microspheres//AC (45.3 W h kg^−1^ at the power density of 533.3 W kg^−1^),^56^ NiCo_2_O_4_/NGN/CNTs//NGN/CNTs (42.7 W h kg^−1^ at the power density of 775 W kg^−1^),^51^ and NiCo_2_O_4_@Co–Fe LDH//AC (28.94 W h kg^−1^ at the power density of 950 W kg^−1^).^52^ Besides NiCo_2_O_4_ based ASC devices, the previously reported ASCs based on other materials have either inferior or equal performance to the presented (NiCo_2_O_4_ sample 2//AC ASC), such as CuS hollow micro flowers//AC (15.97 W h kg^−1^ at a power density of 185.4 W kg^−1^)^57^ and Ni_3_Se_2_ 3D HMNN//AC (38.4 W h kg^−1^ at a power density of 794.5 W kg^−1^).^58^ For simplicity, the observed performance of the ASCs is provided in Table S5.†

### 3.4. Oxygen evolution reaction performance evaluation of various citrus lemon juice assisted NiCo_2_O_4_ nanostructures

**Fig. 5:**
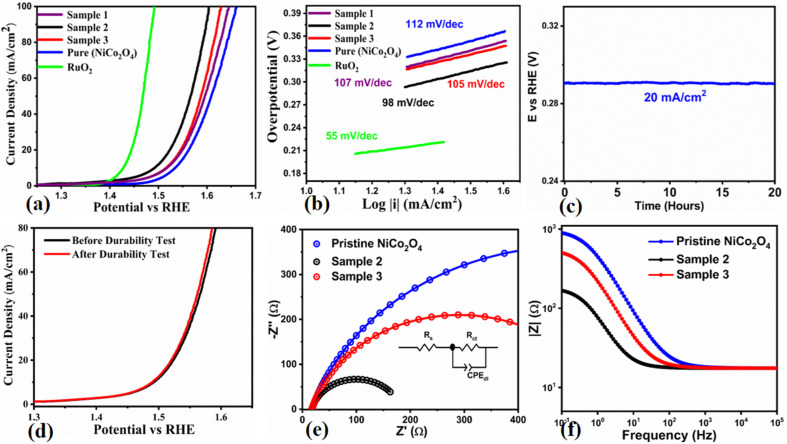
(a) LSV polarization curves at 2 mV s^−1^ of the pristine NiCo_2_O_4_ nanostructures, NiCo_2_O_4_ (sample 2), NiCo_2_O_4_ (sample 3) and RuO_2_ in 1.0 M KOH aqueous solution, (b) corresponding Tafel analysis of the pristine NiCo_2_O_4_ nanostructures, NiCo_2_O_4_ (sample 2), NiCo_2_O_4_ (sample 3) and RuO_2_, (c) stability of NiCo_2_O_4_ (sample 3), (d) durability for 40 hours at 20 mA cm^−2^, and (e) electrochemical impedance spectroscopy (EIS) Nyquist plots of pristine NiCo_2_O_4_ nanostructures, NiCo_2_O_4_ (sample 2), and NiCo_2_O_4_ (sample 3) for the frequency range of 100 kHz to 0.1 Hz at an amplitude of 5 mV and onset potential of OER, inset shows the fitted equivalent circuit. (f) Corresponding Bode Plots of the pristine NiCo_2_O_4_ nanostructures, NiCo_2_O_4_ (sample 2), and NiCo_2_O_4_ (sample 3) from the same EIS data.

The electrochemical performance of the NiCo_2_O_4_ nanostructures was studied for OER half-cell water splitting in 1.0 M KOH aqueous solution using the three electrode cell configuration. For this characterization, LSV was used for the pure NiCo_2_O_4_ and lemon juice assisted NiCo_2_O_4_ nanostructures (sample 1, sample 2, and sample 3) at a sweeping scan rate of 2 mV s^−1^ and the corresponding *iR* corrected polarization curves are provided in Fig. 5a. The performance of these newly prepared materials of NiCo_2_O_4_ was also compared with the reference noble metal (RuO_2_) electrocatalyst, as shown in Fig. 5a. The overpotential for the NiCo_2_O_4_ nanostructures grown with 1 mL of citrus lemon juice was found to be 250 mV at 10 mA cm^−2^, which is smaller than those of the pure and 2 mL citrus lemon juice assisted NiCo_2_O_4_ nanostructures. An overpotential of 270 mV at 10 mA cm^−2^ was recorded for the pure and 2 mL assisted NiCo_2_O_4_ nanostructures, as shown in Fig. 5a. Whereas, sample 1 showed relatively better performance compared to the pure NiCo_2_O_4_ nanostructures with a low overpotential of 280 mV at 10 mA cm^−2^. However, 1 mL of citrus lemon juice drastically changed the OER activity of the NiCo_2_O_4_ nanostructures with a lower overpotential compared to sample 1, sample 2 and the pure NiCo_2_O_4_ nanostructures. The pure NiCo_2_O_4_ nanostructures showed limited performance owing to the low density of active sites and poor electron transfer capacity. Addition of 2 mL of citrus lemon juice supplied a high concentration of reducing agents, which significantly provided the surface with a limited number of active sites and charge transport of the material, hence poor performance of the NiCo_2_O_4_ nanostructures was observed compared to sample 2. The linear region of the LSV polarization curves was used to assess the reaction kinetics and the estimated Tafel plots are given in Fig. 5b. Significantly, the Tafel slope of the NiCo_2_O_4_ nanostructures grown with 1 mL was as low as 98 mV dec^−1^, which is low compared to the pure and 2 mL assisted NiCo_2_O_4_ nanostructured materials (105 mV dec^−1^, and 112 mV dec^−1^, respectively). The Tafel slope of sample 1 was estimated to be about 107 mV dec^−1^. The Tafel analysis revealed that the NiCo_2_O_4_ nanostructures grown with 1 mL of citrus lemon juice have speedy OER kinetics due to their favorable catalytic surface properties. The stability of the 1 mL assisted NiCo_2_O_4_ nanomaterial was also studied in 1.0 M KOH using LSV polarization curves recorded before and after the durability measurements, as shown in Fig. 5c. It was found that the durability test for 40 hours did not alter the onset potential, overpotential and current density, suggesting the high stability and compatibility of the material with the GCE. Durability is an important parameter to examine the activity of a nonprecious catalyst; hence, chronopotentiometry was used to study the electro catalytic durability of the NiCo_2_O_4_ nanostructures prepared with 1 mL of citrus lemon juice, as shown in Fig. 5d. There was negligible overpotential drop at a constant 20 mA cm^−2^ for the period of 40 hours, confirming the durability of the material for a significant period of OER operation. The NiCo_2_O_4_ nanostructures were found to be stable under 1.0 M KOH alkaline conditions and this is strongly supported by reported works elsewhere. It has been observed that the electrochemical active surface area (ECSA) is directly connected to the double layer capacitance (*C*_dl_) of an electrocatalyst,^59^ and it is known as a crucial parameter to assess the amount of active sites on the surface of a catalyst, which are involved in the electrochemical reaction. For this purpose, cyclic voltammetry (CV) curves were measured with non-faradaic regions to calculate the *C*_dl_, as shown in ESI Fig. S7.† The *C*_dl_ values were obtained by plotting the difference of anodic and cathodic current densities at 0.2 V *versus* RHE against different scan rates.^60^ The *C*_dl_ is considered as a half value of slope achieved after linear fitting and ECSA could be measured by using equation ECSA = *C*_dl_/*C*_s_. *C*_s_ is a constant value related to the nature of the electrode material surface and it was found to be 40 mF cm^−2^ in 1.0 M KOH as previously reported.^61^ The measured values of ECSA for the three NiCo_2_O_4_ nanostructures (sample 2, sample 3, and the pure sample) were 15.2 μF cm^−2^, 8.2 μF cm^−2^, and 6.3 μF cm^−2^ respectively. The ECSA calculations revealed that the NiCo_2_O_4_ nanostructures (sample 2) with the highest value exhibited the fastest OER activity. Furthermore, the charge transfer resistance rate was also evaluated for the NiCo_2_O_4_ nanostructures prepared with and without citrus lemon juice during OER operation using electrochemical impedance spectroscopy (EIS), as shown in Fig. 5e. Bode plots obtained from EIS data at different sweeping frequencies are shown in Fig. 5f. The higher frequency region of the Nyquist plot provided information about the porosity of the electrode film whereas the low frequency region of the sub circuit is associated with the charge transfer resistance.^62^ The fitted circuit provided the charge transfer resistance values for the three NiCo_2_O_4_ nanostructures (sample 2, sample 3 and pure) of 165 Ω, 547 Ω and 968 Ω, respectively, and these values are provided in Table S6.† The charge transfer resistance is low for sample 2, further supporting its OER performance in 1.0 M KOH aqueous solution. The XRD results of sample 2 revealed that the preferred crystal orientation is along the 400 crystal plane, which is a desirable crystal orientation for the exposure of high surface catalytically active sites, consequently fast charge transport occurred at the interface of electrode and electrolyte. The HRTEM study showed that NiCo_2_O_4_ (sample 2) is composed of nanoparticles of less than 20 nm forming a large surface area. Therefore, a low nanoscale dimension of less than 20 nm of sample 2 offered frequent contact with electrolyte, thus efficient electrochemical performance was observed. The SAED pattern of the lemon juice assisted NiCo_2_O_4_ (sample 2) presents rings at 2.4 and 2.0 Å, indicating small lattice rings. Hence, these aspects of sample 2 could be driving the electrochemical activity of material towards supercapacitor and half-cell OER applications. The improved performance of the proposed electrocatalyst could be explained from a fundamental point of view in terms of the shape transformation of the material, variation in crystal orientation, high concentration of surface oxygen vacancies, highly active sites, fast charge transfer rate at the interface of electrode and electrolyte favoring the electrocatalytic kinetics, and high Ni/Co ratio of 1.37, and the biomimetic features of the citrus lemon juice assisted NiCo_2_O_4_ sample supported the high compatibility with the glassy carbon electrode. The present study aimed to understand the effects of structural transformation, creation of defects on the surface, and altering the crystal orientation during the growth process, and they together enhanced the electrochemical performance of the NiCo_2_O_4_ nanostructures. These fundamental features of the prepared NiCo_2_O_4_ nanostructures of the present study represent an advancement in the development of a new generation of electrocatalysts for energy conversion and storage systems. Furthermore, the enhanced SC and electrocatalytic performances of the NiCo_2_O_4_ nanostructures (sample 2) were contributed by the unique fabrication aspects, such as short range nanoparticles resulting from the unique chemistry of citrus lemon juice exposing the material to a large extent of electrolytic molecules. The use of biomass has brought the nanoparticles very closely and the volume changes of nanostructured NiCo_2_O_4_ were avoided. Hence, the citrus lemon juice maintained the mechanical strength of NiCo_2_O_4_, and demonstrated the significant cycling stability during SC and OER processes.

The OER performance of the NiCo_2_O_4_ nanostructures (sample 2) prepared with citrus lemon juice was compared with the previously reported electrocatalysts given in Table S7.† It is clear that the activity of the NiCo_2_O_4_ nanostructures (sample 2) is superior to many of the catalysts in terms of a low overpotential of 250 mV at the same current density and in 1.0 M KOH aqueous solution, confirming the dynamic role of natural molecules from citrus lemon juice in the enhanced activity towards water splitting. Importantly, the synthetic strategy of the presented work is green, using minimal toxic chemicals, being low cost, having the potential for scale up for the synthesis of a large quantity of material, and being ecofriendly and environment friendly. Furthermore, the obtained electrochemical performance is shown as a bar graph for better understanding in ESI Fig. S8a–d.† We report the obtained results in these bar graphs for the OER overpotential at 10 and 50 mA cm^−2^, Tafel values, specific capacitance (*C*_s_) and energy density, respectively, for the pristine and citrus lemon juice assisted NiCo_2_O_4_ nanostructures (sample 1, sample 2, and sample 3) and RuO_2_. For a better view, the OER data of sample 1, sample 2, and sample 3 and capacitance performance of the pure and citrus lemon juice assisted NiCo_2_O_4_ nanostructures (sample 2 and sample 3) are given in Table 1. It is clear that sample 2 has relatively better electrochemical activity compared to the pure NiCo_2_O_4_ and sample 3.

**Table 1:** Overall performance comparison of the pristine and citrus lemon juice assisted NiCo_2_O_4_ nanostructures (sample 1 and sample 2)

Sample ID	Overpotential (mV) @ 10 mA cm^−2^	Tafel slope (mV dec^−1^)	Charge transfer resistance (*R*_ct_) (Ω)	Specific capacitance (F g^−1^) @ 0.8 A g^−1^	Energy density (W h kg^−1^) @ 0.8 A g^−1^	Power density (W kg^−1^) @ 0.8 A g^−1^
Pure (NiCo_2_O_4_)	300	112	968	117	2.47	156
Sample 1	280	107	—	—	—	—
Sample 2	250	98	165	358	7.95	160
Sample 3	280	105	547	163	3.57	159

## 4. Conclusions

In summary, we have used natural resources of chemical compounds with stabilizing, capping and chelating properties from citrus lemon juice for the preparation of nanoparticles of NiCo_2_O_4_ using a hydrothermal method. The structure, chemical composition and crystalline studies were carried out by using a wide range of analytical techniques. We have observed that the nanorod morphology of NiCo_2_O_4_ was successfully transformed into nanoparticles due to a change in pH of the growth solution, and the capping agent, stabilizing agent and chelating agent properties of citric acid, malic acid and ascorbic acid during the growth process. At the same time, these natural products tailored the surface properties, and enhanced electron communication, fast ionic diffusion of electrolyte, and swift charge transfer at the interface of electrode and electrolyte. For this reason, we have noticed an excellent OER reaction at an overpotential of 250 mV at 10 mA cm^−2^ with a durability of 40 hours under alkaline conditions. Furthermore, we have also studied the energy storage aspects of the NiCo_2_O_4_ material for the development of asymmetric supercapacitors and observed values of specific capacitance (*C*_s_) of 1519.19 F g^−1^ and energy density of 33.08 W h kg^−1^. The extended spectrum of electrochemical properties of the NiCo_2_O_4_ material in addition to our low cost and facile approach are highly favorable for the development of high performance energy conversion and storage systems in the near future.

## Author contributions

Shusheel Kumar, carried out material synthesis and water catalysis; Aneela Tahira, did XRD analysis; Adeel Liaquat Bhatti, did impedance measurement; Muhammad Ali Bhatti, did OER analysis; Riaz Hussain Mari, did partial supervision; Nek Muhammad Shaikh, did partial supervision; Muhammad Yameen Solangi, did ECSA and Tafel analysis; Ayman Nafady, previewed the obtained results; Mélanie Emo, did TEM analysis; Brigitte Vigolo, did SEM analysis and proofread the paper; Antonia Infantes-Molina, did XPS analysis and proofread the paper; Alberto Vomiero, did the final preview and proofread the paper; Zafar Hussain Ibupoto, did supervision and wrote the first draft of the manuscript.

## Conflicts of interest

Authors declare no conflict of interest in this research work.

## Supplementary Material

RA-013-D3RA02438E-s001

## References

[cit1] Yan J., Wang Q., Wei T., Fan Z. (2014). Adv. Energy Mater..

[cit2] Poonam K. S., Arora A., Tripathi S. K. (2019). J. Energy Storage.

[cit3] Gou J., Du Y., Xie S., Liu Y., Kong X. (2019). Int. J. Hydrog. Energy.

[cit4] Raman V., Mohan N. V., Balakrishnan B., Rajmohan R., Rajangam V., Selvaraj A., Kim H. J. (2020). Ionics.

[cit5] Wang F., Li Y., Cheng Z., Xu K., Zhan X., Wang Z. (2014). Phys. Chem. Chem. Phys..

[cit6] Gou J., Xie S., Xu B. (2020). Ionics.

[cit7] Liu L., Zhang H., Yang J., Mu Y., Wang Y. (2015). J. Mater. Chem. A.

[cit8] Fu H., Liu Y., Chen L., Shi Y., Kong W., Hou J., Yu F., Wei T., Wang H., Guo X. (2019). Electrochim. Acta.

[cit9] Liu T., Diao P. (2020). Nano Res..

[cit10] Kumar L., Chauhan M., Boruah P. K., Das M. R., Hashmi S. A., Deka S. (2020). ACS Appl. Energy Mater..

[cit11] Zhu Y., Ji X., Wu Z., Song W., Hou H., Wu Z., He X., Chen Q., Banks C. E. (2014). J. Power Sources.

[cit12] Gao X., Zhang H., Li Q., Yu X., Hong Z., Zhang X., Liang C., Lin Z. (2016). Angew. Chem., Int. Ed..

[cit13] Mondal A., Maiti S., Mahanty S., Panda A. B. (2017). J. Mater. Chem. A.

[cit14] Fang L., Jiang Z., Xu H., Liu L., guan Y., Gu X., Wang Y. (2018). J. Catal..

[cit15] Lv X., Zhu Y., Jiang H., Yang X., Liu Y., Su Y., Huang J., Yao Y., Li C. (2015). Dalton Trans..

[cit16] Hosseini S. E., Wahid M. A. (2020). Int. J. Energy Res..

[cit17] Perera F. (2018). Int. J. Environ. Res. Publ. Health.

[cit18] Elavarasan R. M. (2020). IEEE Access.

[cit19] Olabi A., Abdelkareem M. A. (2022). Renew. Sustain. Energy Rev..

[cit20] Gielen D., Boshell F., Saygin D., Bazilian M. D., Wagner N., Gorini R. (2019). Energy Strategy Rev..

[cit21] Wang Q., Domen K. (2019). Chem. Rev..

[cit22] Jamesh M.-I., Sun X. (2018). J. Power Sources.

[cit23] Zhang Q., Guan J. (2020). J. Power Sources.

[cit24] Cao L. M., Li L., Wang P., Shao Q. (2018). Adv. Sci..

[cit25] Suen N.-T., Hung S.-F., Quan Q., Zhang N., Xu Y.-J., Chen H. M. (2017). Chem. Soc. Rev..

[cit26] Chen Z., Duan X., Wei W., Wang S., Ni B.-J. (2020). Nano Energy.

[cit27] Fabbri E., Habereder A., Waltar K., Kötz R., Schmidt T. J. (2014). Catal. Sci. Technol..

[cit28] Matsumoto Y., Sato E. (1986). Mater. Chem. Phys..

[cit29] Liang Q., Chen J., Wang F., Li Y. (2020). Coord. Chem. Rev..

[cit30] Lu Z., Li L., Wang P., Shao Q. (2014). Nat. Commun..

[cit31] Gao L., Cui X., Wang Z., Sewell C. D., Li Z., Liang S., Zhang M., Li J., Hu Y., Lin Z. (2021). Proc. Natl. Acad. Sci. U. S. A..

[cit32] Gao L., Cui X., Sewell C. D., Li J., Lin Z. (2021). Chem. Soc. Rev..

[cit33] Vij V., Sultan S., Harzandi A. M., Meena A., Tiwari J. N., Lee W. G., Yoon T., Kim K. S. (2017). ACS Catal..

[cit34] Khalafallah D., Zhi M., Hong Z. (2021). ChemCatChem.

[cit35] Kaur M., Chand P., Anand H. (2021). Inorg. Chem. Commun..

[cit36] Sasmal A., Nayak A. K. (2023). J. Energy Storage.

[cit37] Pore O. C., Fulari A. V., Velhal N. B., Parale V. G., Park H. H., Shejwal R. V., Fulari V. J., Lohar G. M. (2021). Mater. Sci. Semicond. Process..

[cit38] Yedluri A. K., Kim H. J. (2019). RSC Adv..

[cit39] Kaur M., Chand P., Anand H. (2021). Inorg. Chem. Commun..

[cit40] Solangi A. G., Pirzada T., Shah A. A., Halepoto I. A., Chang A. S., Solangi Z., Solangi M. Y. (2022). J. Chin. Chem. Soc..

[cit41] Shafi P. M., Joseph N., Karthik R., Shim J.-J., Bose A. C., Ganesh V. (2021). Microchem. J..

[cit42] Poonguzhali R. V., Li L., Wang P., Shao Q. (2021). Ceram. Int..

[cit43] Klimek-Szczykutowicz M., Szopa A., Ekiert H. (2020). Plants.

[cit44] Laghari A. J., Ibhupoto Z., Bhatti A. L. (2022). Int. J. Hydrogen Energy.

[cit45] Bhatti A. L., Ibhupoto Z., Tahira A., Aftab U. (2021). Electrochim. Acta.

[cit46] Aftab U., Laghari A. J., Ibhupoto Z., Bhatti A. L. (2019). Catal. Sci. Technol..

[cit47] Lu Y.-T., Chien Y.-J., Liu C.-F., You T.-H., Hu C.-C. (2017). J. Mater. Chem. A.

[cit48] Tong Y. L., Xing L., Dai M. Z., Wu X. (2019). Front. Mater..

[cit49] Lei D. Y., Li X. D., Seo M. K., Khil M. S., Kim H. Y., Kim B. S. (2017). Polymer.

[cit50] Pu J., Wang J., Jin X. Q., Cui F. L., Sheng E., Wang Z. H. (2013). Electrochim. Acta.

[cit51] Wu Y. Q., Chen X. Y., Ji P. T., Zhou Q. Q. (2011). Electrochim. Acta.

[cit52] Iqbal N., Wang X. F., Babar A. A., Yu J. Y., Ding B. (2016). J. Colloid Interface Sci..

[cit53] Chang L., Mai L., Xu X., An Q., Zhao Y., Wang D., Feng X. (2013). RSC Adv..

[cit54] Xu Z., Ren J., Meng Q., Zhang X., Du C., Chen J. (2019). ACS Sustain. Chem. Eng..

[cit55] Liu L., Zhang H., Yang J., Mu Y., Wang Y. (2015). J. Mater. Chem. A.

[cit56] Liu Y., Zhou Z., Zhang S., Luo W., Zhang G. (2018). Appl. Surf. Sci..

[cit57] Liu Y., Xu Q., Wang R., Zheng Y., Zhu L., Wang Z., Zheng W. (2020). J. Mater. Chem. A.

[cit58] Zhang G., Qin Q., Luo W., Liu Y., Jin C., Hao J., Zhang J., Zheng W. (2018). Chem. Commun..

[cit59] Xu Q., Liu Y., Tian Z., Shi Y., Wang Z., Zheng W. (2021). Electrochim. Acta.

[cit60] Li W., Gao X., Xiong D., Wei F., Song W. G., Xu J., Liu L. (2017). Adv. Energy Mater..

[cit61] Birry L., Lasia A. (2004). J. Appl. Electrochem..

[cit62] Liao L., Wang S., Xiao J., Bian X., Zhang Y., Scanlon M. D., Hu X., Tang Y., Liu B., Girault H. H. (2014). Energy Environ. Sci..

