# Supplementary material for: Transforming NiCo2O4 nanorods into nanoparticles using citrus lemon juice enhancing electrochemical properties for asymmetric supercapacitor and water oxidation
Source: RSC Adv. 2023 Jun 20;13(27):18614–26. doi: 10.1039/d3ra02438e (PMC10280130; doi:10.1039/d3ra02438e)
Supplement: RA-013-D3RA02438E-s001 [file RA-013-D3RA02438E-s001.pdf]

Electronic Supplementary Material (ESI) for RSC Advances.
This journal is © The Royal Society of Chemistry 2023

Supporting Information

Transforming $NiCo_2O_4$ nanorods into nanoparticles using citrus lemon juice enhancing electrochemical properties for asymmetric supercapacitor and water oxidation

Shusheel Kumar[a], Aneela Tahira[c], Adeel Liaquat Bhatti[a], Muhammad Ali Bhatti[d], Riaz Hussain Mari[a], Nek Muhammad Shaikh[a], Muhammad Yameen Solangi[e], Ayman Nafady[j], Mélanie Emo[f], Brigitte Vigolo[f], Antonia Infantes-Molina[g], Alberto Vomiero*[h,i], Zafar Hussain Ibupoto*[b]

[a]Institute of Physics, University of Sindh Jamshoro, 76080, Sindh Pakistan

[b]Institute of Chemistry, University of Sindh Jamshoro, 76080, Sindh Pakistan

[c]Institute of Chemistry, Shah Abdul Latif University Khairpur Mirs, Sindh, Pakistan

[d]Institute of Environmental Sciences, University of Sindh Jamshoro, 76080, Sindh Pakistan

[e]Mehran University of Engineering and Technology, 7680 Jamshoro, Sindh Pakistan

[f]Université de Lorraine, CNRS, IJL, F-54000 Nancy, France

[g]Department of Inorgnic Chemistry, Crystallography and Mineralogy. (Unidad Asociada al ICP-CSIC), Faculty of Sciences, University of Malaga, Campus de Teatinos, 29071, Malaga, Spain

[h]Department of Engineering Sciences and Mathematics, Division of Material Science, Luleå University of Technology, Luleå, Sweden

[i]Department of Molecular Sciences and Nanosystems, Ca' Foscari University of Venice, Venezia Mestre, Italy

[j]Chemistry Department, College of Science, King Saud University, Riyadh, 11451, Saudi Arabia

***Corresponding authors**: Alberto Vomiero and Zafar Hussain Ibupoto

Email: alberto.vomiero@unive.it, zaffar.ibhupoto@usindh.edu.pk

Calculation (S2): Calculation of supercapacitor of 1D $NiCo_2O_4$ nanorods and its 0D nanoparticles

The calculations of Specific Capacitance C_s and energy density was done according to the published work [1] using the following mathematical relations:

$$C_s = \frac{I \times \Delta t}{m \times \Delta V}$$

Specific capacitance is C_s, I as current, Δt discharge time, ΔVpotential range, m mass of deposited material onto GCE

Whereas energy density was calculated as:

$$E_d = \frac{C_s \times (\Delta V^2)}{2}$$

E_d as energy density, speiifc caapciatnec as C_s, ΔV^2 change in potential

Power density was measured as follows [1]:

$$P_d = \frac{E}{\Delta t}$$

P_d represents the power density; E is the energy and Δt discharge time.

Asymmetric supercapacitor was fabricated using two electrodes set up involving positive electrode $NiCo_2O_4$ nanostructures prepared with 2 mL of lemon juice and negative electrode of activated carbon (AC) in 3.0M KOH electrolytic solution. Commercial activated carbon was used for the development of negative electrode during two electrode configuration for the asymmetric device system. The slurry of activated carbon was prepared by dispersing 17 mg of Ac into 3:1 volume of ethanol and the deionized water. In this mixture 100µL of 5% Nafion was also added. The well dispersed slurry was achieved by sonication for 10 min. The anode of $NiCo_2O_4$ and cathode of AC was developed onto GCE respectively. The asymmetric supercapacitor was described by following configuration of $NiCo_2O_4$ sample 2//AC ASC. The balancing of voltammetric charges for the prepared $NiCo_2O_4$ sample 2 and AC was done with calculation of masses of two electrode by following relationship as previously reported Q+ = Q– equation [2].

$m+ \ m- = C_s- \ \Delta V- \ C_s+ \Delta V+$.

Here: m±, Cs±, and ΔV± describes the mass, specific capacitance C_s, and potential range of $NiCo_2O_4$ sample 2 (+) and AC (−) electrodes, respectively. The estimated total mass of m+/m− was about 0.17 mg in $NiCo_2O_4$ sample 2//AC ASC. For the calculation of C_s, and energy density and power density the total mass was calculated as per the reported work [3].

$$C_s = \frac{4 \times I \times \Delta t}{m \times \Delta V}$$

C_s represents specific capacitance, I current, discharge time Δt, potential change ΔV, loaded mass as m.

Energy density was calculated as:

$$E_d = \frac{C_s \times (\Delta V^2)}{7.2}$$

E_d is energy, C_s as specific capacitance, potential variation ΔV^2

The power density was determined by [2]:

$$P_d = \frac{C_s \times 3600}{\Delta t}$$

Here P_d is the power density, C_s is the specific capacitance and the discharge time is Δt

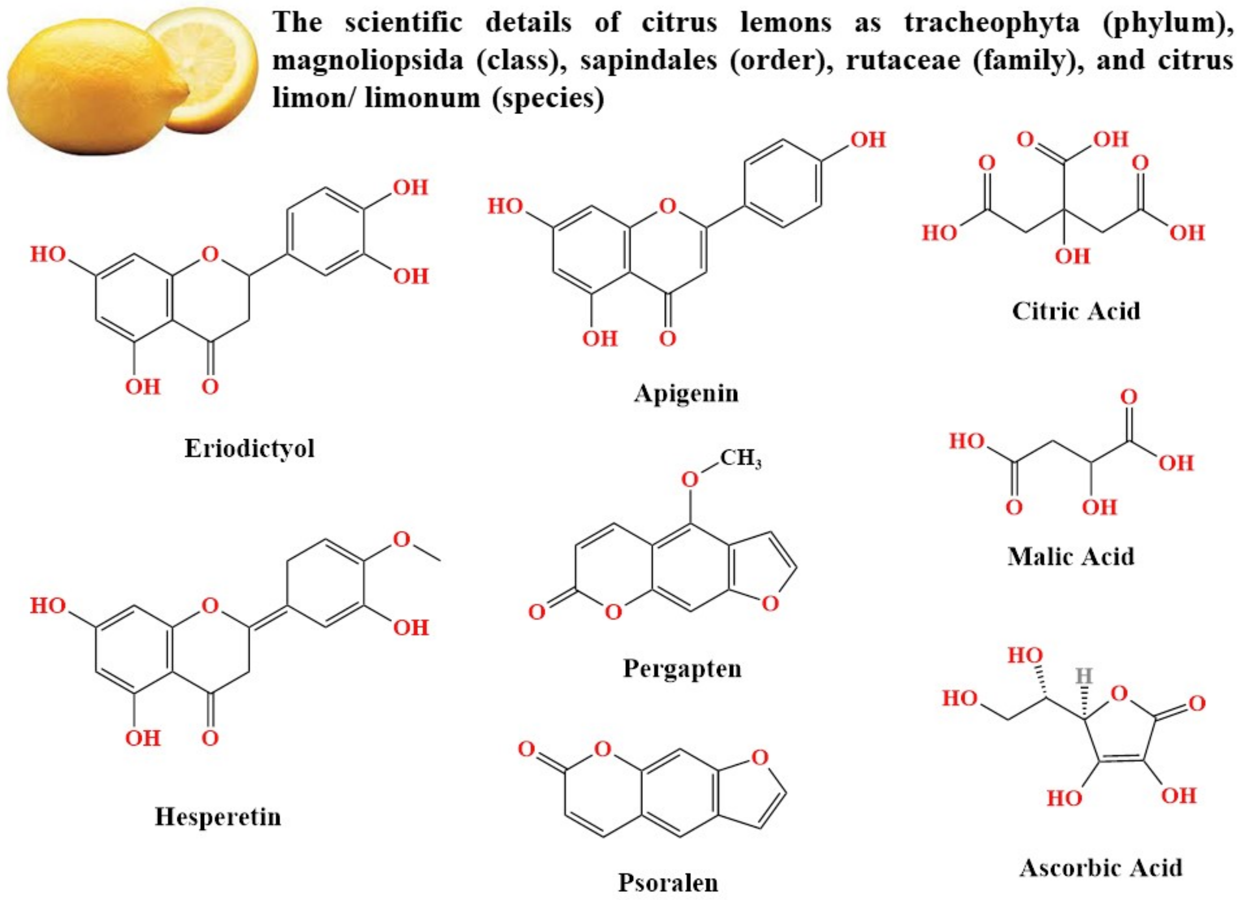

Scheme (S1): Chemical compounds in the lemon juice.

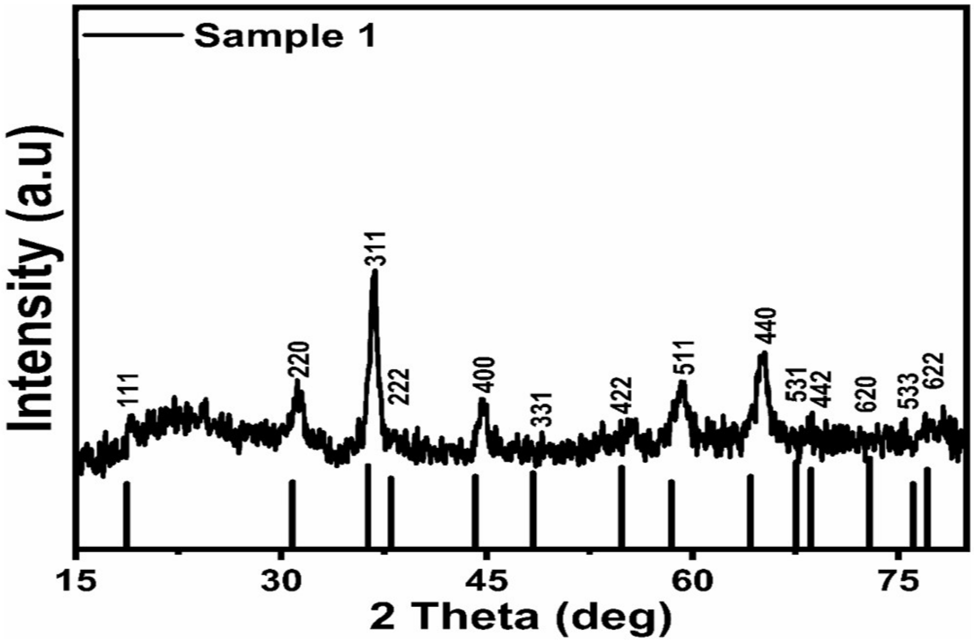

Figure S1: XRD diffraction patterns of sample 1 prepared with 0.5 mL of citrus lemon juice

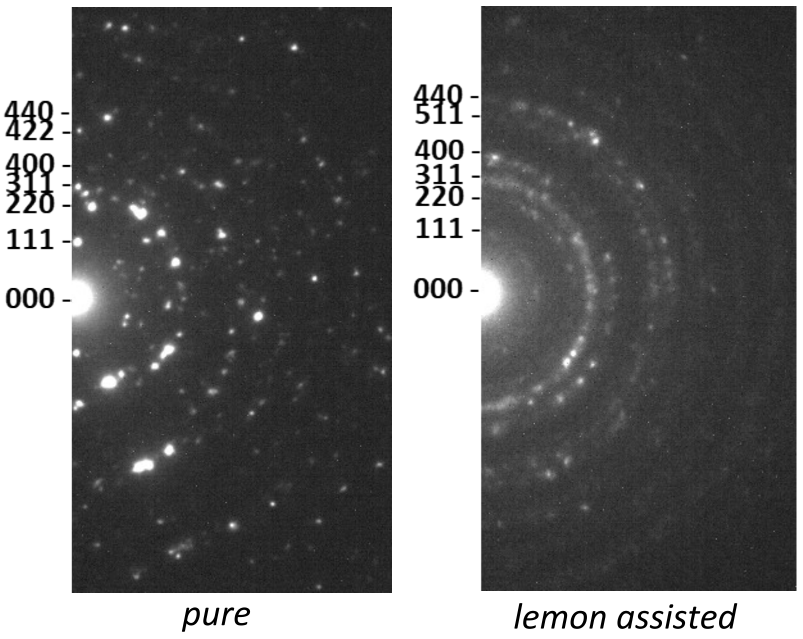

Figure S2: SAED patterns of pure $NiCo_2O_4$ and lemon assisted $NiCo_2O_4$ nanostructures

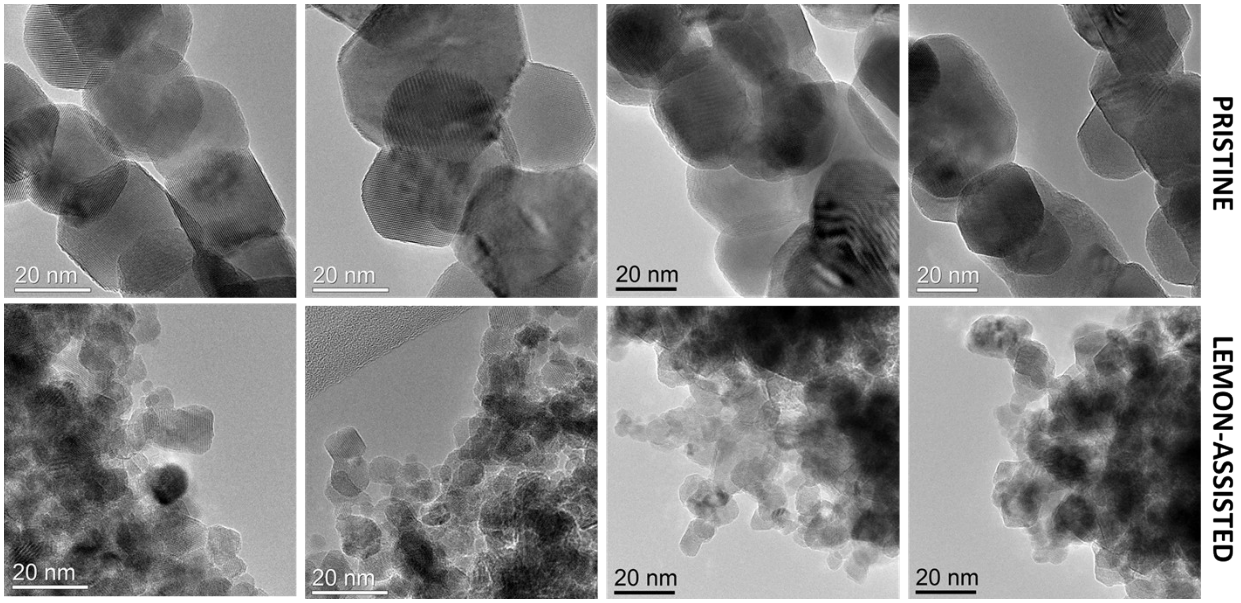

Figure S3: TEM micrographs of pure (top) and lemon-assisted (bottom) $NiCo_2O_4$

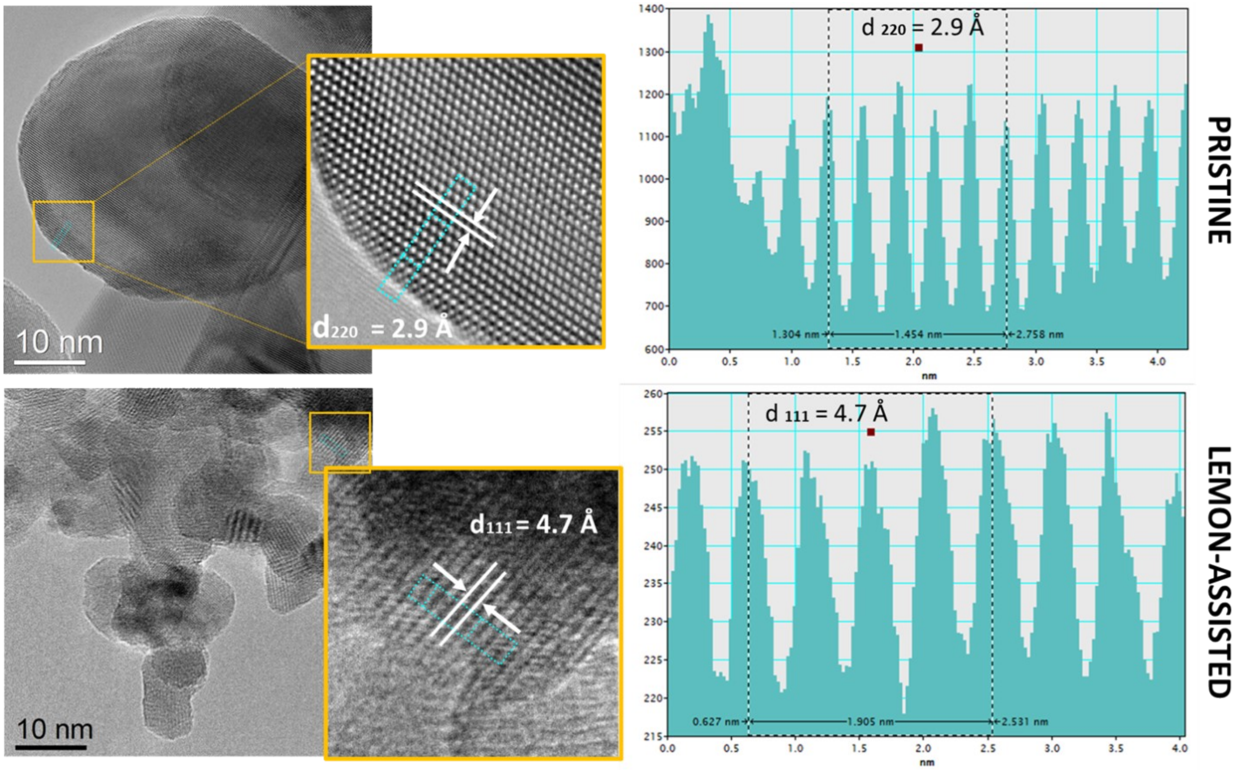

Figure S4: HRTEM micrographs of pure (top) and lemon-assisted (bottom) $NiCo_2O_4$ with corresponding intensity profiles taken in blue squared area of HRTEM images

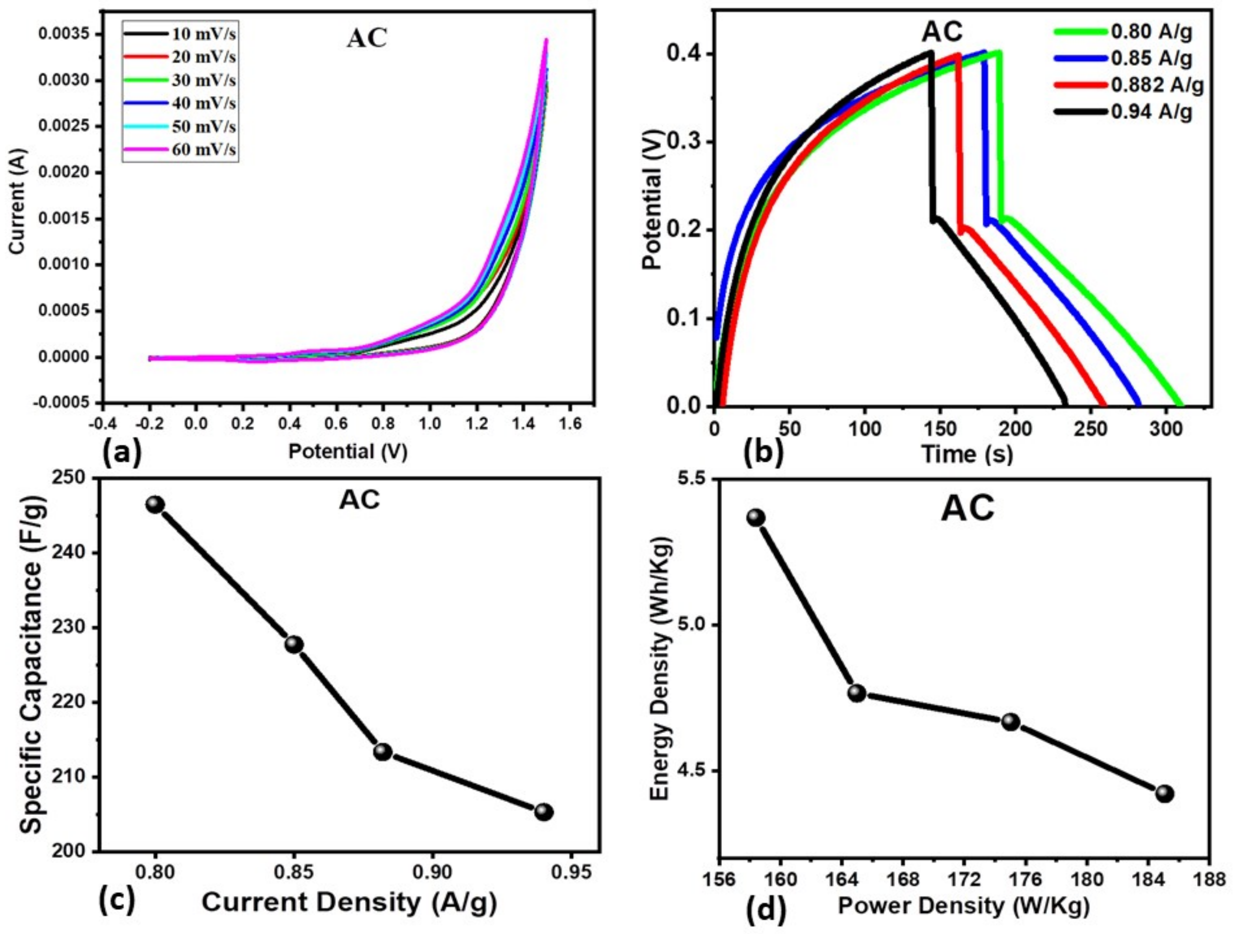

Figure S5: Various CV curves of activated carbon (AC) at various scan rates in 3.0M KOH, (b) GCD curves at different current density for AC, (c) specific capacitance of AC, (d) energy density and power density calculated values of AC

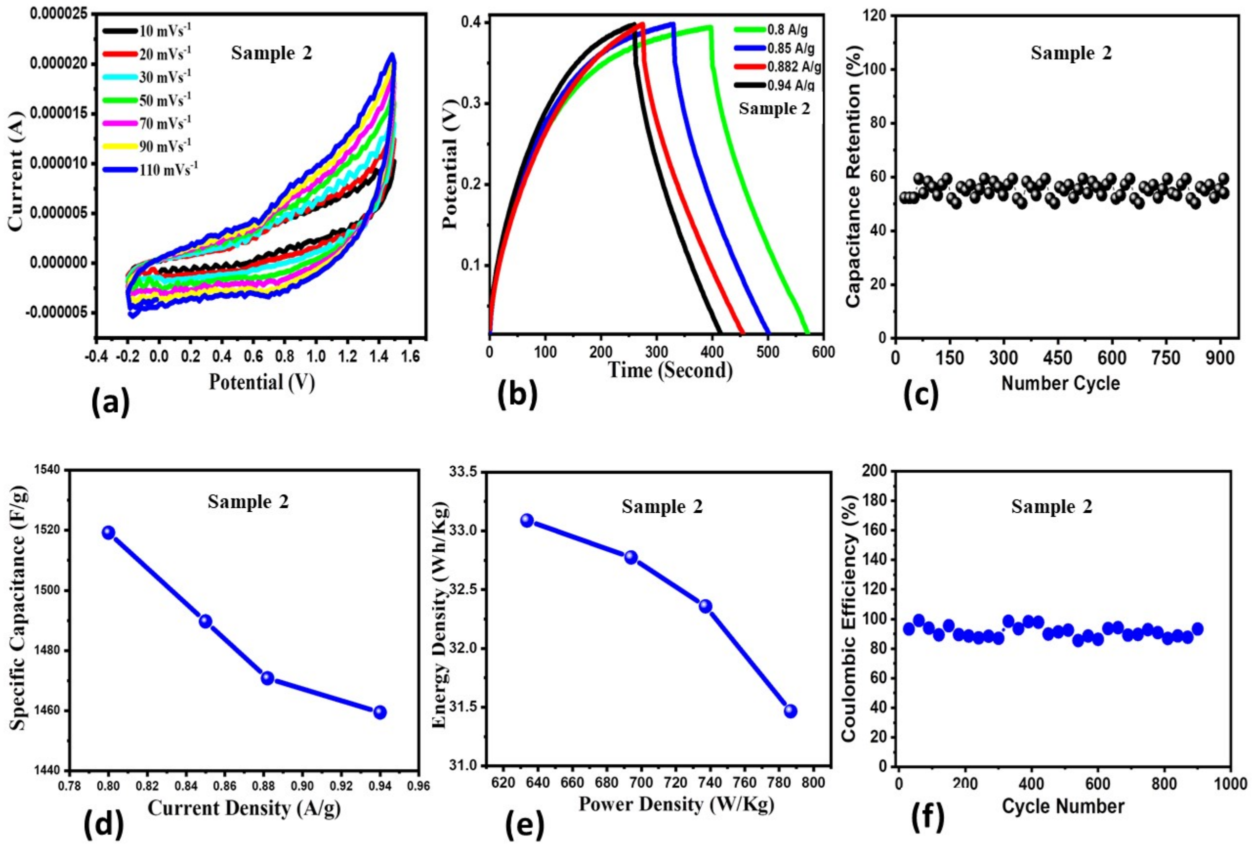

Figure S6: Various CV curves of $NiCo_2O_4$ (sample 2) at different scan rates in 3.0M KOH, (b) GCD curves at different current density for $NiCo_2O_4$ (sample 2), (c) Cycling stability of $NiCo_2O_4$ (sample 2), (d) specific capacitance of $NiCo_2O_4$ (sample 2) from GCD curves, (e) Energy density of $NiCo_2O_4$ (sample 2), (f) Columbic efficiency of $NiCo_2O_4$ (sample 2) during cycling stability.

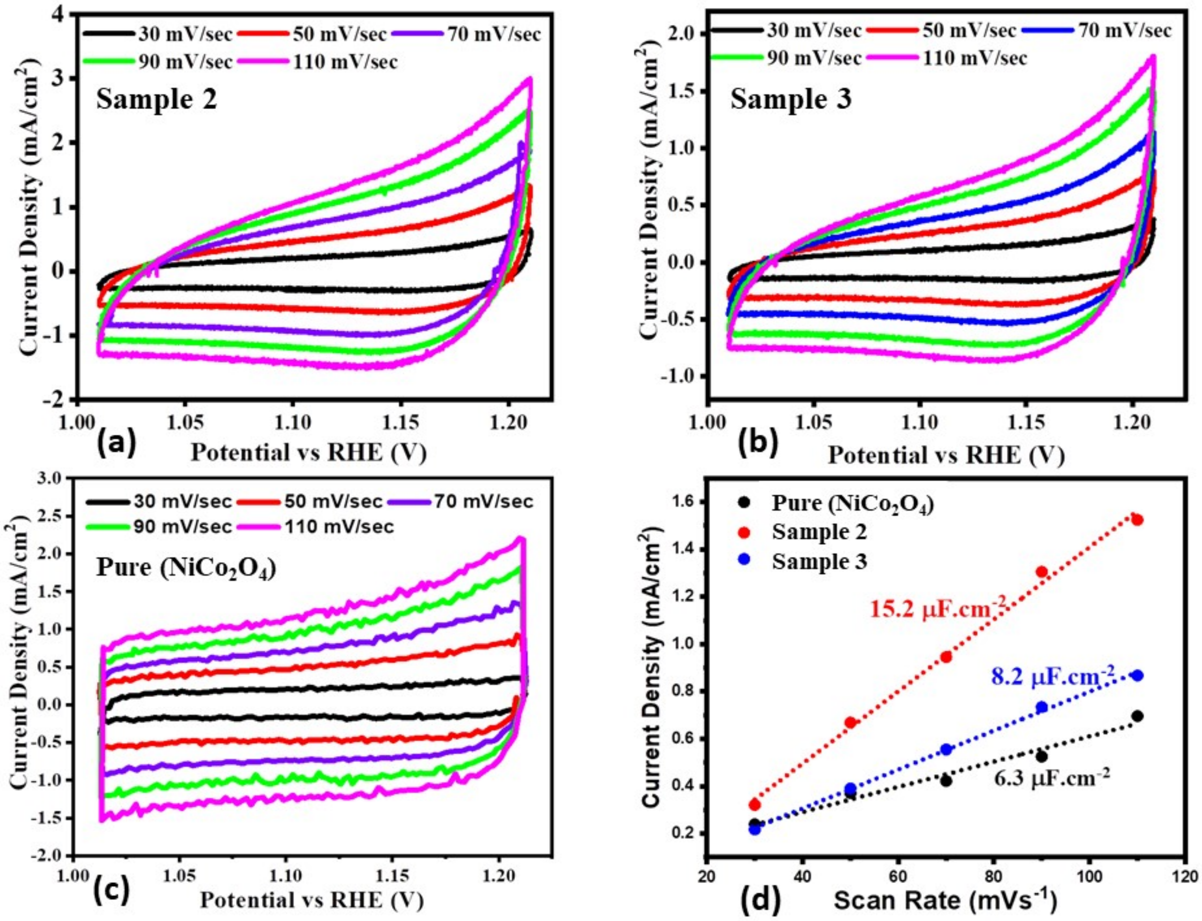

Figure S7: Different CV curves at variosu scan rates in 1.0M KOH for (a) pristine $NiCo_2O_4$ nanostructure, (b) $NiCo_2O_4$ (sample 2), (c) $NiCo_2O_4$ (sample 3), (d) caluclated ECSA from non-Faradic region of estimated CV curevs for pristine $NiCo_2O_4$ nanostructure, $NiCo_2O_4$ (sample 2), $NiCo_2O_4$ (sample 3).

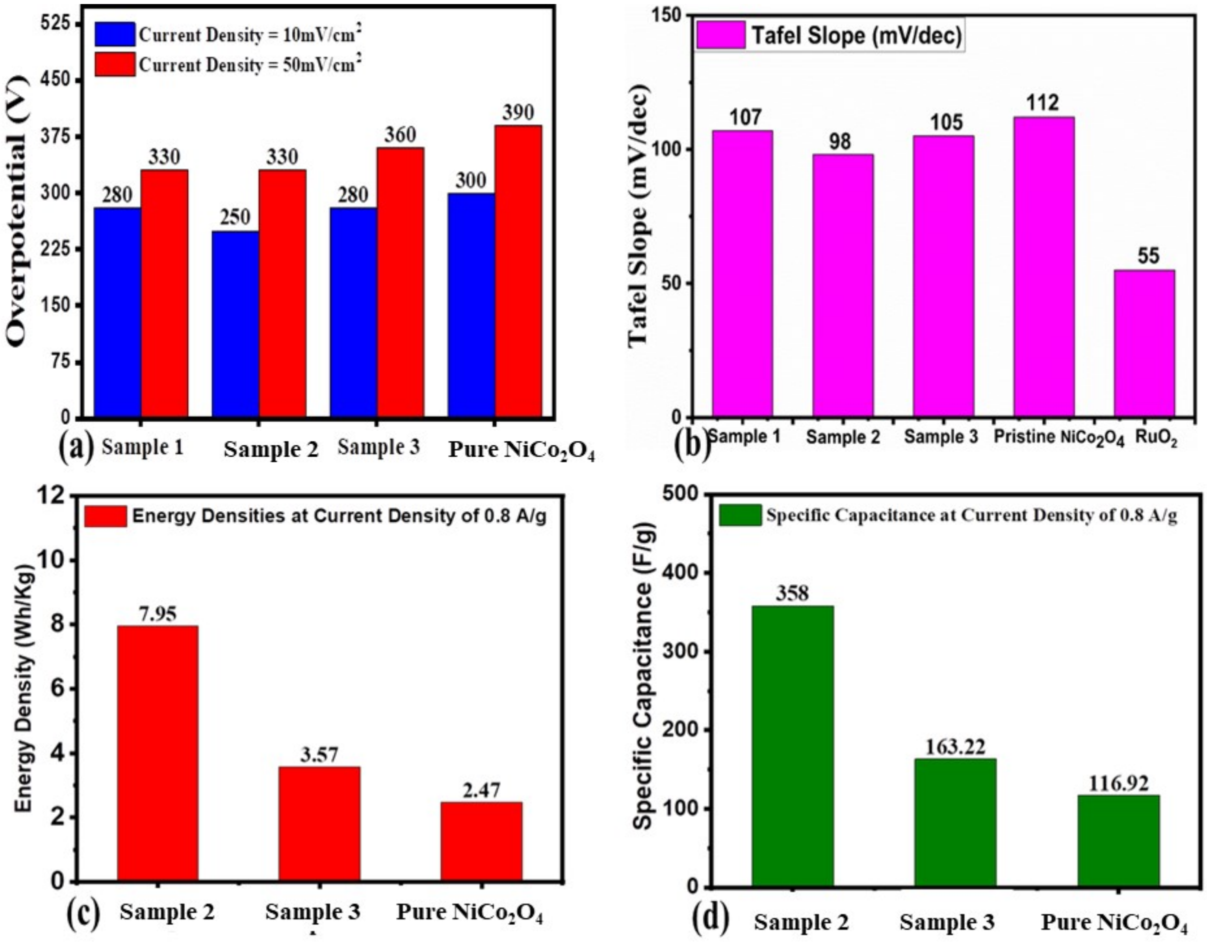

Figure S8: Bar graph view (a) overpotential at 10 and 50 mAcm^{-2} for RuO_2, pure $NiCo_2O_4$ nanostructure, $NiCo_2O_4$ (sample 2), $NiCo_2O_4$ (sample 3), (b) Tafel slope for RuO_2, pristine $NiCo_2O_4$ nanostructure, $NiCo_2O_4$ (sample 2), $NiCo_2O_4$ (sample 3), (c) Energy density of supercapacitor for pristine $NiCo_2O_4$ nanostructure, $NiCo_2O_4$ (sample 2), $NiCo_2O_4$ (sample 3), (d) specific capacitance (Cs) for pristine $NiCo_2O_4$ nanostructure, $NiCo_2O_4$ (sample 2) and (sample 2).

Table S1: Calculated average crystallite size of pure $NiCo_2O_4$ and sample 1, sample 2 and sample 3.

**Sample ID**	**Height**	**FWHM**	**Crystalline size (nm)**
Pure $NiCo_2O_4$	44.61	0.26	14
Sample 1	36.84	0.28	16
Sample 2	45.03	0.25	14
Sample 3	44.06	0.12	18

Table (S2): Detailed XPS peak attributions for pure $NiCo_2O_4$ and sample 2

Pure ($NiCo_2O_4$)

Ni 2p3/2								
Band	Pos	PosSep	B_FWHM	FWHM	Height	%Gauss	Area	%Area
1	854.03	0	1.8	1.8	2730	90	5480	28.34
2	855.75	1.73	1.86	1.86	2442	100	4830	24.98
3	857.02	3	2.08	2.08	644	90	1492	7.72
4	861.11	7.08	4.68	4.68	1379	80	7532	38.96

Co 2p3/2								
Band	Pos	PosSep	B_FWHM	FWHM	Height	%Gauss	Area	%Area
1	779.54	0	2.2	2.2	6105	80	15681	54.97
2	781.39	1.85	2.24	2.24	2627	80	6855	24.03
3	784.21	4.67	4.55	4.55	780	100	3781	13.25
4	788.93	9.39	4	4	495	90	2212	7.76

O 1s								
Band	Pos	PosSep	B_FWHM	FWHM	Height	%Gauss	Area	%Area
1	529.43	0	1.11	1.11	9630	80	12480	61.02
2	531.13	1.7	1.54	1.54	3977	100	6509	31.82
3	532.69	3.26	1.6	1.6	820	90	1464	7.16

Sample 2

Ni 2p3/2								
Band	Pos	PosSep	B_FWHM	FWHM	Height	%Gauss	Area	%Area
1	853.95	0	1.8	1.8	2230	100	4273	29.14
2	855.67	1.73	1.8	1.8	1889	100	3619	24.69
3	856.9	2.95	1.7	1.7	521	100	944	6.44
4	860.85	6.9	5.5	5.5	978	96	5825	39.73

Co 2p3/2								
Band	Pos	PosSep	B_FWHM	FWHM	Height	%Gauss	Area	%Area
1	779.29	0	2.02	2.02	3274	100	7045	53.88
2	781.09	1.8	2.3	2.3	1722	80	4617	35.31
3	784.07	4.79	4.5	4.5	192	100	918	7.02
4	788.81	9.52	4.58	4.58	101	100	494	3.78

O 1s								
Band	Pos	PosSep	B_FWHM	FWHM	Height	%Gauss	Area	%Area
1	529.25	0	1.2	1.2	6083	90	8139	47.59
2	531.08	1.83	1.82	1.82	3078	90	6234	36.45
3	532.53	3.27	1.5	1.5	1562	80	2731	15.96

Table S3: The calculated various supercapacitor indicators pure $NiCo_2O_4$ nanostructure (sample 2), $NiCo_2O_4$ (sample 3)

Samples	Current Density (Ag^{-1})	Specific Capacitance (Fg^{-1})	Energy Density (Wh kg^{-1})	Power Density (W kg^{-1})	Columbic Efficiency (%)	Capacitance Retention (%)
Sample 2	0.8	358	8	160	70%	100-94%
	0.85	284	6	167		
	0.882	126	3	176		
	0.94	118	3	188		
Sample 3	0.8	163	4	159	64%	108-83%
	0.85	154	3	169		
	0.882	149	3	175		
	0.94	60	1	183		
Pure ($NiCo_2O_4$)	0.8	117	2	156	43%	90-61%
	0.85	113	2	166		
	0.882	112	2	171		
	0.94	109	2	183		

Table S4: Supercapacitor performance evaluation of as synthesized $NiCo_2O_4$ nanostructure with lemon juice (sample 2) with reported supercapacitor based on $NiCo_2O_4$

Material	Specific Capacitance (Fg^{-1})	Current Density (Fg^{-1})	Potential Window	Energy Density (Wh kg^{-1})	Power Density (W kg^{-1})	Reference
NCO@MWCNT	374 F/g	2 A/g	-0.5 to 2.2V	95	3964	4
MWCNTs	84 F/g	0.6 A/g	-0.5 to 2.2V	21	6237	4
NCO//MWCNT	157 F/g	0.6 A/g	-0.5 to 2.2V	40	2816	4
NCO@MWCNT//MWCNT	242 F/g	0.6 A/g	-0.5 to 2.2V	61	2837	4
NiCoF	50.0	1 A/g	0 to 1 V	-	-	5
NiCuF	44	1 A/g	0 to 1 V	-	-	5
NC6	1294.25	10 A/g	0.4	-	-	6
NC10	687.20	10 A/g	0.4	-	-	6
Ni–Co–O–1	568	20 A g	0 to 0.5 V	19.72	5000	7
Ni–Co–O–2	532	20 Ag	-	-	-	7
**$NiCo_2O_4$ (Lemon juice)**	**358**	**0.8 A/g**	**0 to 0.4 V**	**7.96**	**160**	**This Work**

Table S5: Measured indicators for asymmetric supercapacitor using two electrode configuration of $NiCo_2O_4$ (sample 2) for practical applications

Samples	Current Density (Ag^{-1})	Specific capacitance (Fg^{-1})	Energy density (Wh kg^{-1})	Power Density (W kg^{-1})	Columbic Efficiency (%)	Capacitance Retention (%)
Sample 2	0.8	1519	33	634	100-84%	64-52% (900 Cycles)
	0.85	1490	33	694		
	0.882	1471	32	737		
	0.94	1459	31	787		

Table S6: Fitting values of EIS for charge transfer and double layer capacitance for pristine $NiCo_2O_4$ nanostructure, $NiCo_2O_4$ (sample 2), $NiCo_2O_4$ (sample 3)

Samples	Charge Transform Resistance (Rct in Ω)	Double Layer Capacitor (Cdl in Farad)
Pristine $NiCo_2O_4$	968	0.32
Sample 2	165	2.28
Sample 3	547	0.74

Table S7: The summary of OER activity performance of lemon juice assisted $NiCo_2O_4$ nanostructures with recently reported electrocatalysts in 1.0 M KOH.

Catalyst	Over potential (mV) @ 10 mA/cm^2	Electrolyte	References
$NiCo_2O_4$@Graphene Nano sheets	383	1.0 M KOH	8
NiOx/$NiCo_2O_4$/Co_3O_4	315	1.0 M KOH	9
$NiCo_2O_4$/CoO/graphite	323	1.0 M KOH	10
MOF derived-$NiCo_2O_4$/NiO	430	1.0 M KOH	11
P-doped $NiCo_2O_4$ on Ni-Foam	300	1.0 M KOH	12
Co_8FeS_8/CoS@CNT	278	1.0 M KOH	13
CoSx/MoS_2	347	1.0 M KOH	14
$NiCo_2O_4$/NiO	360	1.0 M KOH	15
$Co_xNi_{1-x}S_2$ (CNS)/RGO	290	1.0 M KOH	16
3D core–shell $NiCo_2O_4$@CoS/NF	290	1.0 M KOH	17
CoFe/Co_8FeS_8/CNT	290	1.0 M KOH	18
$NiCo_2O_4$/NiO hexagonal rods	285	1.0 M KOH	19
Ni_3S_2/MoS_2 (NiMoS)	260	1.0 M KOH	20
Ni_3N-NiMoN	277	1.0 M KOH	21
Co_3O_4@CMC	290	1.0 M KOH	22
**Lemon juice assisted $NiCo_2O_4$ material**	**250**	**1.0 M KOH**	**Present Work**

References

[1] E. Jokar & A. zad & S. Shahrokhian, J Solid State Electrochem 2015, 19, 269–274.

[2] Y. Liu, Y. Zheng, Q. Xu, Y. Shi, Z. Tian, R. wang, G. Zhang, J. Chen, Z. Wang, W. Zheng, Chem. Eng. J. 2020, 387, 12412-21.

[3] C.Young, R. R. Salunkhe, S. M. Alshehri,T. Ahamad, Z. Huang, J. Henzie, and Y. Yamauchi, J. Mater. Chem. A, 2017, 5, 11834-11839.

[4] M. Pathak, J. R. Jose, B. Chakraborty, and C. S. Rout, The Journal of Chemical Physics, 2020, 152, 064706.

[5] B. Bhujun, M. T. Tan, and A. S. Shanmugam, Results in Physics, 2017, 7, 345-353.

[6] M. Kaur, P. Chand, and H. Anand, Journal of Energy Storage, 2022, 52, 104941.

[7] H. Wang, Q. Gao, and L. Jiang, small, 2011, 7, 2454-2459.

[8] Z. Li, B. Li, J. Chen, Q. Pang, P. Shen. Int. J. Hydrogen Energy., 2019, 44, 16120–16131.

[9] J. Chen, Y. Ling, Z. Lu, X. Huai, F. Qin, Z. Zhang. Electrochim. Acta. 2019, 322, 134753.

[10] N. Srinivasa, L. Shreenivasa, P.S. Adarakatti, J.P. Hughes, S.J. Rowley-Neale, C.E. Banks, S. Ashoka, RSC Adv. 2019, 9, 24995–25002.

[11] Y. Wang, Z. Zhang, X. Liu, F. Ding, P. Zou, X. Wang, Q. Zhao, H. Rao. ACS Sustainable Chem. Eng. 2018, 6, 12511–12521.

[12] W. Chu, Z. Shi, Y. Hou, D. Ma, X. Bai, Y. Gao, N. Yang. ACS Appl. Mater. Interfaces, 2020, 12, 2763–2772.

[13] B. Wang, Y. Chen, X. Wang, X. Zhang, Y. Hu, B. Yu, D. Yang, W. Zhang. J. Power Sources. 2020, 449, 227561.

[14] L. Yang, L. Zhang, G. Xu, X. Ma, W. Wang, H. Song, D. Jia. ACS Sustainable Chem. Eng. 2018, 6, 12961–12968.

[15] C. Mahala, M. Basu. ACS Omega 2017, 2, 7559–7567.

[16] Y.-R. Hong, S. Mhin, K.-M. Kim, W.-S. Han, H. Choi, G. Ali, K.Y. Chung, H.J. Lee, S.-I. Moon, S. Dutta, S. Sun, Y.-G. Jung, T. Song, H. Han. J. Mater. Chem. A 2019, 7, 3592–3602.

[17] S. Adhikari, Y. Kwon, and D. H. Kim. Chemical Engineering Journal, 2020, 402, 126192.

[18] B. Wang, Y. Hu, B. Yu, X. Zhang, D. Yang, Y. Chen. J. Power Sources., 2019, 433, 126688.

[19] Y. Yuan, L. Sun, Y. Li, W. Zhan, X. Wang, X. Han, Inorg. Chem. 2020, 59, 4080–4089.

[20] C. Wang, X. Shao, J. Pan, J. Hu, X. Xu. Appl. Catal. B., 2020, 268, 118435.

[21] A. Wu, Y. Xie, H. Ma, C. Tian, Y. Gu, H. Yan, X. Zhang, G. Yang, and H. Fu. Nano Energy, 2018, 44, 353-363.

[22] A. L. Bhatti, A. Tahira, A. Gradone, R. Mazzaro, V. Morandi, M.I. Abro, A. Nafady, K. Qi. Electrochimica Acta, 2021, 398, 139338.
